# High Entropy Alloys Mined From Binary Phase Diagrams

**DOI:** 10.1038/s41598-019-50015-4

**Published:** 2019-10-29

**Authors:** Jie Qi, Andrew M. Cheung, S. Joseph Poon

**Affiliations:** Department of Physics, University of Virginia, Charlottesville, VA 22904-4714 USA

**Keywords:** Computational science, Materials science, Condensed-matter physics, Structural materials, Theory and computation

## Abstract

High entropy alloys (HEA) are a new type of high-performance structural material. Their vast degrees of compositional freedom provide for extensive opportunities to design alloys with tailored properties. However, compositional complexities present challenges for alloy design. Current approaches have shown limited reliability in accounting for the compositional regions of single solid solution and composite phases. For the first time, a phenomenological method analysing binary phase diagrams to predict HEA phases is presented. The hypothesis is that the HEA structural stability is encoded within the phase diagrams. Accordingly, we introduce several phase-diagram inspired parameters and employ machine learning (ML) to classify 600+ reported HEAs based on these parameters. Compared to other large database statistical prediction models, this model gives more detailed and accurate phase predictions. Both the overall HEA prediction and specifically single-phase HEA prediction rate are above 80%. To validate our method, we demonstrated its capability in predicting HEA solid solution phases with or without intermetallics in 42 randomly selected complex compositions, with a success rate of 81%. The presented search approach with high predictive capability can be exploited to interact with and complement other computation-intense methods such as CALPHAD in providing an accelerated and precise HEA design.

## Introduction

High entropy alloys were first discovered in 2004^1,2^. They are also known as multi principal elements (MPE) alloys or compositionally complex alloys (CCA). HEAs can form as either single or mixed phases. HEAs have emerged as one of the most popular topics in material research^1–5^. These materials span vast compositional space, providing flexibility in alloy design^6–11^. However, the compositional complexity poses a significant challenge in the control of phase formation due to thermodynamic and kinetic constraints^12,13^. Empirical approaches that utilised atomistic and thermodynamic parameters^3,14–16^ were first introduced to investigate the compositional regions of HEA phases, but with only limited success. Additionally, first-principles calculation^16–21^ and Calculation of Phase Diagrams (CALPHAD)^22^ methods have been employed to shed light on the atomistic and thermodynamic mechanisms of HEA formation. However, the accuracy of CALPHAD is often limited by the availability of thermal databases, and the appearance of miscibility gaps and intermetallic (IM) compounds in the binary systems^23^. Monte Carlo simulations show promising results in predicting the formation of certain IM phases and the phase structure evolution with varying temperatures^24^. Employing statistical approaches, a thermodynamics and Gaussian process statistical model^25^ that utilised up to nine parameters was proposed as the basis for identifying single solid solution phases. Another model using a database of over 2000 multicomponent alloy compositions from a high-throughput sputter deposition experiment, applied a regression method to interrogate the HEA phase formation tendency^26^. Models using neural networks were trained based on atomistic and thermodynamic parameters to predict the HEA phase categories without specific phase formation information^27,28^. Despite recent progress in understanding the formation trend of subgroups of HEAs, the constitution of HEAs still relies on trial and error, which impedes the design of these multicomponent alloys for fundamental studies and applications.

High entropy provides the driving force for a HEA system to form a single solid solution phase. A distinctive feature of good HEA forming systems is significant to moderate solid solution formation tendency among the constituent binary alloys. However, the experimental scenario is more complicated. Rather than forming single solid solution phase HEAs, different solid solution phases can coexist with occasionally IM formation occurring. The phase formation and stability can be influenced by temperature and atomic interactions, such as a miscibility gap or atomic level strain. Except for the computation-intensive studies, atomic interactions are usually not comprehensively accounted for by the prior mentioned models. On the other hand, the experimentally validated phase diagrams are encoded with the binary atomic interaction information.

Departing from current approaches, we present herein a phenomenological method using binary phase diagrams to predict the compositional space of HEA phases. The advantage of using binary phase diagrams to assess phase stability is that they can readily provide direct and realistic information about the roles of individual elemental components on phase formation. The phenomenological method is built on the hypothesis that the constituent binary alloys encode a wealth of information about the multi-component alloy in terms of crystal structures, elemental mixing, and phase separation. Here, we demonstrate the effectiveness of the proposed method by introducing physically meaningful phenomenological parameters that can be conveniently accessed from binary phase diagrams. These parameters are used to demarcate the phases forming regions for HEAs. The phases studied here are those with homogeneity ranges in the phase diagrams such as body-centred cubic (BCC) single-phase, face-centred cubic (FCC) single-phase, mixed FCC + BCC phase, hexagonal close-packed (HCP) single-phase, Sigma phase, and Laves phase. Minor phases such as line compounds are not included but will be for future work. ML algorithm is employed to navigate the complex parameter space regions occupied by the currently known HEA compositions. The effectiveness of the method is evaluated, and the derived ML algorithms are used to make predictions for experimental verification. The presented “phase diagram” approach to predicting single solid solution HEAs can complement CALPHAD and other first-principles methodologies in providing an efficient pathway to phase-field and microstructural control.

## Database Partitioning

The HEAs included in our model have phases classified as: disordered FCC (A1), disordered BCC (A2), disordered HCP (A3), mixed disordered FCC + BCC (A1 + A2), ordered BCC (B2), B2 mixed with disordered solid solution phases specifically A1, A2, and A3 (B2 + SS), and either Sigma or Laves IM mixed with the other phases (IM+). The set of HEAs included in A1 + A2 are the commingling of A1s, A2s, or the coexistence of A1s and A2s. The set of HEAs included in the IM+ phase have at least Sigma or Laves phase. Additionally, the IM+ phase may also contain other complex or solid solution phases. The database is parsed into three different levels, namely, Level 1, 2, and 3. Level 1 is composed of the simple disordered phases: A1, A2, A1 + A2, and A3. Level 2 is Level 1 with the addition of the B2 + SS HEAs. And Level 3 is Level 2 with the addition of IM+ HEAs. HEAs with other minor phases such as line compounds that do not belong to the above categories are not included in the present study. Levels 1, 2, and 3 comprise 317, 486, and 614 HEAs respectively. More details about the database can be found in the method section and the supplementary materials.

## HEA Phase Formation Parameters

The parameters, introduced below, and elaborated on in detail in the method section, provide the basis for quantifying HEA phase formation tendencies. For ML, these individually measured property parameters, used as input data to do classification, are called features.

The HEA melting temperature (T_m_) is expressed as the weighted average of binary liquidus temperatures. For the as-cast HEAs, undercooling usually extends to the region around 0.8 T_m_^29^. Phase evolution may still exist below this temperature because of the high kinetic energies of the atoms. Here, a phase formation temperature (T_pf_) is defined where rapid phase evolution ceases. It is assumed that T_pf_ is not lower than 0.7 T_m_. Below this temperature, the kinetic energy of atoms is not high enough to transform the phase within the brief time of cooling. Incidentally, most post-annealed HEAs in the full database are homogenised above 0.7 T_m_. Atoms are free to exchange neighbours during undercooling (i.e. above 0.8 T_m_), or via fast diffusion down to T_pf_. The alloy mixture is essentially ergodic and local atoms have nearly equal probabilities of sampling any binary configurations favoured by the phases present in the constituent binary phase diagrams.

Following the above discussion, information from individual binary phase diagrams is combinatorially used within the model. It is assumed that the tendency for a pair of elements to form a specific phase is directly determined by its binary phase field percentage. The binary phase field percentage of phase X for i-j elemental pair is denoted as ${{\rm{X}}}_{{\rm{i}}-{\rm{j}}}$ and is determined using T_pf_. Then, ${{\rm{X}}}_{{\rm{i}}-{\rm{j}}}$ is used to calculate the phase field parameter (PFP_X_) which is related to the tendency of a HEA to form a phase X.

Many mixed phase HEAs are found to form because of interatomic repulsions^30,31^. Specific element pairs, such as Cr and Cu, separate because of the large positive mixing enthalpy, causing multiphase formations in HEAs^30^. This effect is included in the model with the phase separation parameter (PSP).

The selection of T_pf_ can influence the values of parameters and the prediction accuracy. The optimized T_pf_ value was obtained to get the most accurate ML prediction. Further details for T_pf_ determination and calculating these parameters (value ≤ 1) are found in the method section.

## Visualisation of Phase Regions in Parameter Space

The prior defined parameters are calculated for all HEAs in different database levels. Their correlations with the actual phases formed are examined.

**Figure 1:**
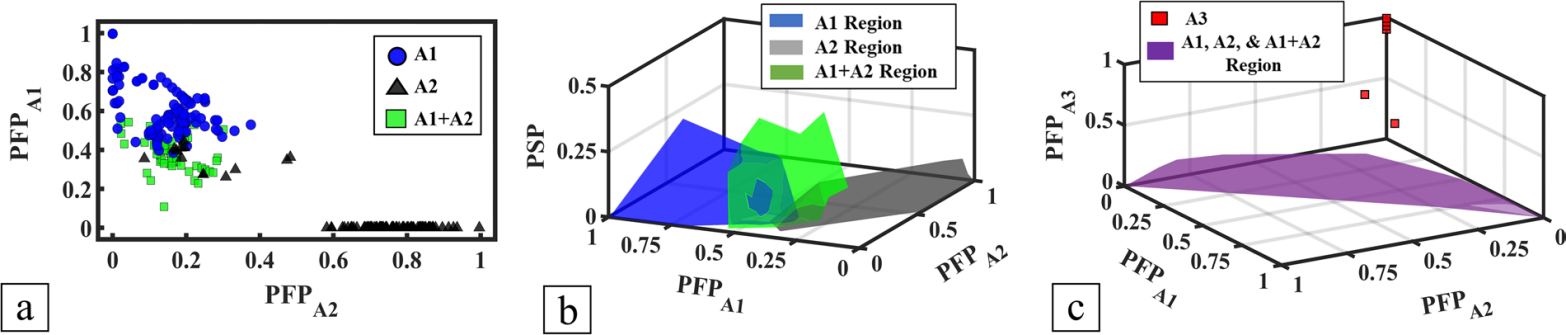
Visualisations of Level 1 HEA parameters PFP_A1_, PFP_A2_, PFP_A3_, and PSP for phases A1, A2, A3, and A1 + A2. (**a**) PFP_A1_ is plotted against PFP_A2_ for A1, A2, and A1 + A2 HEAs; (**b**) PFP_A1_, PFP_A2_, and PSP are plotted for phase regions of A1, A2, and A1 + A2 HEAs; and (**c**) PFP_A1_, PFP_A2_, and PFP_A3_ are plotted for A3 HEAs and phase region of non-A3 (A1, A2, and A1 + A2) HEAs.

For the Level 1 phases, there are correlations between the calculated parameters PFP_A1_, PFP_A2_, PFP_A3_, and PSP with the A1, A2, A3, and A1 + A2 phase formation. Figure 1a, a plot of PFP_A1_ verse PFP_A2_ shows the parameters partitioning the A1, A2, and A1 + A2 HEAs. Typically, A1 HEAs have PFP_A1_ > 0.4 and PFP_A2_ < 0.4, while A2 HEAs have PFP_A1_ < 0.4. Some A2 HEAs form even with small PFP_A2_ values because their B2 phase field can transfer into the A2 phase to prompt A2 formation. As discussed in the following Level 2 and Level 3, specific phase formation is influenced by multiple parameters. A1 + A2 HEAs are distributed in a region where neither PFP_A1_ nor PFP_A2_ is dominant and cannot be separated from the single phase HEAs. In general, individually large PFP_A1_ or PFP_A2_ values promote the formation of a single phase, while the similar values of PFP_A1_ and PFP_A2_ tend to favour a mixed phase formation. Adding PSP as the third axis in Fig. 1b separates the A1 + A2 from the A1 and A2 HEAs by their relative higher PSP values because a large PSP indicts the strong phase separation effect which leads to the A1 + A2 phase formation. To study the effect of PFP_A3_ on A3 phase formation, a plot with axes PFP_A1_, PFP_A2_, and PFP_A3_ is plotted for A1, A2, A3, and A1 + A2 HEAs in Fig. 1c, where A1, A2, and A1 + A2 HEAs are grouped as non-A3 HEAs. All the A3 HEAs have higher PFP_A3_ than the non-A3 HEAs and appear separate from the other phases.

**Figure 2:**
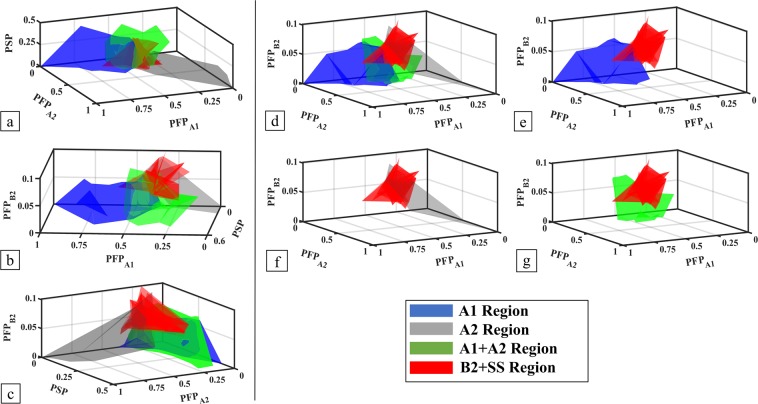
Visualisation of Level 2 parameters PFP_A1_, PFP_A2_, PFP_A3_, PFP_B2_, and PSP for the A1, A2, A1 + A2, and B2 + SS HEA phase regions. (**a**) PFP_A1_, PFP_A2_, and PSP; (**b**) PFP_A1_, PFP_B2_, and PSP; (**c**) PFP_A2_, PFP_B2_, and PSP; (**d**) PFP_A1_, PFP_A2_, and PFP_B2_; and (**e**–**g**) the decomposition of the plot (**d**) highlighting the location of the B2 + SS phase region relative to the A1, A2, and A1 + A2 phase regions.

For the Level 2 phases, the five parameters are PFP_A1_, PFP_A2_, PFP_A3_, PFP_B2_, and PSP. In Fig. 2a–g, to study the effects of the new parameter PFP_B2_, the 5D parameter space of the Level 2 data is visualised by projecting it on to 3D spaces. Figure 2a is plotted with only the parameters in Level 1. B2 + SS HEAs are mixed with HEAs in other phases. In Fig. 2b–d, PFP_B2_ is added. Figure 2e–g have the same axes as Fig. 2d but can give direct comparisons between the B2 + SS phase and the A1, A2, and A1 + A2 phases. On all these plots, B2 + SS HEAs are located in a region with relatively higher PFP_B2_ values. This indicates that PFP_B2_ is strongly correlated with the B2 + SS phase formation. PFP_A3_ and A3 HEAs are not plotted here because PFP_A3_ does not affect the formation of B2 + SS phase and A3 HEAs are trivial to predict with PFP_A3_ as shown in Level 1.

**Figure 3:**
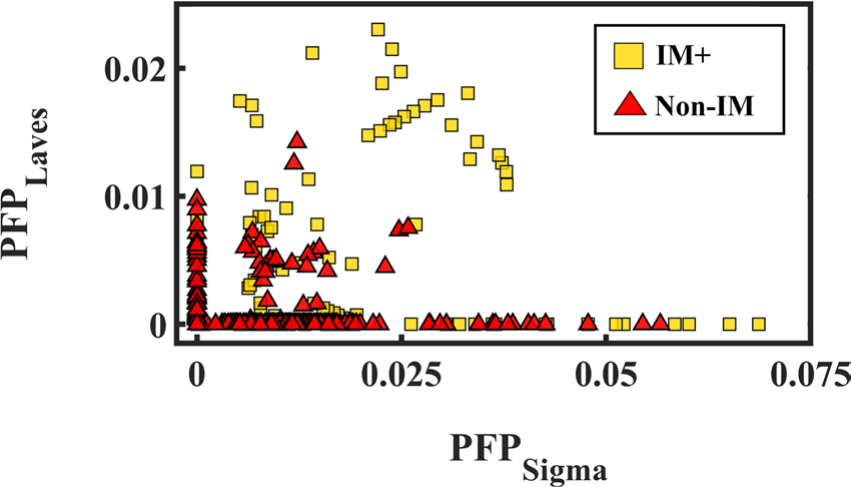
Level 3 parameters PFP_Sigma_ and PFP_Laves_ plotted for IM+ and Non-IM HEAs, where Non-IM includes A1, A2, A3, A1 + A2, and B2 + SS.

For the Level 3 phases, two additional parameters PFP_Sigma_ and PFP_Laves_ are added. Seven parameters PFP_A1_, PFP_A2_, PFP_A3_, PFP_B2_, PFP_Sigma_, PFP_Laves_, and PSP are used to separate the phase regions of A1, A2, A3, A1 + A2, B2 + SS, and IM + HEAs. In order to study the correlation between the newly added IM+ phase formation and the two parameters PFP_Sigma_ and PFP_Laves_, a 2D graph with axes PFP_Sigma_ and PFP_Laves_ is plotted in Fig. 3. All the phases from Level 2 are grouped as Non-IM phases. In general, IM+ HEAs have larger PFP_Laves_ or PFP_Sigma_ than most of the Non-IM HEAs. However, all seven parameters have influence on the IM+ phase formation. Figure 3 is insufficient to convey all the information from the seven parameters.

In summary, Level 1 shows separation between all single phase HEAs in the PFP_A1_, PFP_A2_, PFP_A3_, and PSP parameter space. A1 + A2 phase region is seen to have some overlaps with A1 and A2 phase regions. By adding parameters in Level 2 and Level 3, additional overlaps are noted. The parameter space of the HEAs assumes an increasingly complex topological configuration as the number of parameters increases. Additionaly, it is difficult to resolve the connections in 3D space. In such complicated cases, ML is superior to the visualisation method to determine phase formation regions.

## HEA Phases Prediction Using Machine Learning

ML is employed to analyse the complex parameter space of HEA phase formation. It creates links between the parameters and phase formation in the higher-dimensional parameter space. Through ML composition-phase correlations are determined and new HEA compositions are predicted.

The effect of phase formation from alloy preparation methods is also studied. ML is first applied to only the as-cast HEAs and its performance serves as a benchmark. Then ML is applied to all HEAs in as-cast and annealed states. The ML prediction performance comparison of the HEA sets yields on average that the addition of the annealed HEAs has a slight abating effect, as seen in Table 1.

**Table 1:** ML results and count of HEAs for the three levels of the study.

ML Prediction Success Rate (%)								
		As-Cast [As-Cast + Annealed]						
Level	Training Set Percentage (%)	Phase Category						
		Overall	A1	A2	A1-A2	A3	B2+SS	IM+
Level 1	90							
	80	89 [89]	88 [91]	95 [94]	80 [76]	100 [98]		
	75	90 [88]	89 [90]	95 [93]	81 [73]	100 [100]		
	67	89 [87]	87 [89]	95 [93]	79 [73]	100 [98]		
	50	89 [87]	87 [89]	95 [93]	80 [74]	98 [98]		
Level 2	90	85 [85]	84 [87]	94 [94]	69 [62]	100 [100]	86 [87]	
	80	84 [85]	83 [87]	94 [93]	67 [62]	100 [98]	85 [86]	
	75	85 [84]	84 [86]	94 [93]	68 [60]	100 [98]	86 [85]	
	67	85 [83]	83 [85]	94 [92]	68 [59]	100 [96]	85 [85]	
	50	83 [84]	79 [85]	94 [92]	64 [61]	100 [95]	84 [85]	
Level 3	90	82 [80]	81 [81]	92 [87]	64 [61]	100 [100]	84 [85]	78 [77]
	80	81 [79]	80 [80]	91 [87]	63 [58]	100 [100]	84 [83]	78 [76]
	75	81 [79]	80 [80]	91 [87]	63 [59]	100 [100]	84 [83]	76 [74]
	67	80 [78]	80 [80]	91 [85]	63 [60]	100 [100]	83 [82]	74 [73]
	50	78 [77]	78 [77]	90 [86]	62 [56]	100 [98]	80 [81]	73 [71]
**Total Alloy Counts**								
		**As-Cast [As-Cast + Annealed]**						
		**Phase Category**						
**Level**		**Overall**	**A1**	**A2**	**A1-A2**	**A3**	**B2+SS**	**IM+**
Level 1		235 [317]	69 [118]	101 [121]	61 [72]	4 [6]		
Level 2		375 [486]	69 [118]	101 [121]	61 [72]	4 [6]	140 [169]	
Level 3		470 [614]	69 [118]	101 [121]	61 [72]	4 [6]	140 [169]	95 [128]

ML prediction success rates for the as-cast HEAs and the as-cast + annealed HEAs in different phases are listed. The success rates are F1 scores. Counts of HEAs and phases for the as-cast HEAs and the as-cast + annealed HEAs in different phases are listed.

The ML results for Level 1 HEAs are obtained using the features PFP_A1_, PFP_A2_, PFP_A3_, and PSP. The overall success rates with 50–90% training sets are 89–90% for the as-cast HEAs or 87–89% including the annealed HEAs. Single phase predictions have higher success rates than the mixed phase predictions. The high prediction success rates prove that these parameters are sufficient for describing the disordered solid solution phase formation behaviour. PFP_B2_ is added as a fifth ML feature to predict the B2 + SS HEAs in Level 2. The overall and the B2 + SS phase prediction success rates are near 85% for both the as-cast HEA set and the set including the annealed HEAs. Thus, PFP_B2_ is useful in predicting the presence of the B2 + SS phase. Formation of the IM+ phases in the Level 3 HEAs are studied by adding PFP_Sigma_ and PFP_Laves_ as new features. The IM+ phase prediction success rates are 73–78% for the as-cast HEAs or 71–77% including the annealed HEAs. The overall success rate is as high as 80% for all HEAs.

With the increasing complexity of the database from Level 1 to Level 3, the ML prediction success rates decrease but still maintain high values. As the training set percentages change from 90% to 50% in each level, the success rates show little variance. High accuracy is obtained even with training set percentages as low as 50%.

## Model Validation

**Figure 4:**
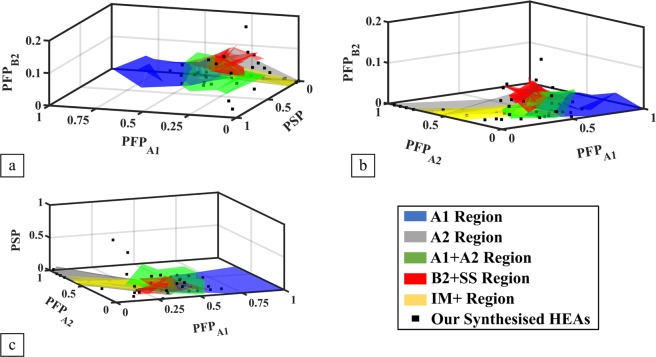
Our synthesised HEAs locations are plotted relative to the phase regions of Level 3 for (**a**) PFP_A1_, PFP_B2_, and PSP; (**b**)PFP_A1_, PFP_A2_ and PFP_B2_; and (**c**) PFP_A1_, PFP_A2_, and PSP parameters.

To show that the model avoids overfitting with ML and can expand the current phase regions, 42 new HEAs were synthesised. The phases of these elemental combinations, which do not exist in the current collected database, are then predicted by the model. As shown in Fig. 4, the selection of compositions is distributed evenly in the parameter space of the collected database. The numbers of new HEAs in different predicted phases are approximately proportional to the numbers of different HEA phases in the database. Many synthesised HEAs are outside the current known phase regions in order to show the ability to expand the phase region. As shown in Table 2, our method is not limited by the use of a specific element type nor the number of elements in a HEA. Elements are chosen from different groups of the periodic table such as refractory metals, transition metals, and main group elements. The number of elements in a single HEA varies from four to seven. All the phases are measured in the as-cast state. Out of the 42 HEAs, 34 were predicted by ML correctly, yielding a success rate of 81%. Their X-Ray Diffraction (XRD) patterns are found in the supplementary materials.

**Table 2:** HEAs synthesised to validate the ML model. The compositions, predicted phases by the ML in Level 3, and the XRD measured phases are listed.

Alloy (at.%)	Predicted Phase	Real Phase	Alloy (at.%)	Predicted Phase	Real Phase
Ag_0.2_Al_2_CrMnNi	A1 + A2	**B2** + **A1** + **A2**	CoCrCuFe	A1 + A2	A1 + A2
AgAlCrMnNi	B2 + SS	B2 + A1 + A2	CoCrCuFeMnNiTi_0.4_	A1 + A2	**A1**
Al_0.2_CoCr_0.5_Fe_2_NiTi_0.25_	A1	A1	CoCrCuMn_0.8_Ti	IM+	Laves + A1
Al_0.2_Cr_1.5_Cu_1.5_Fe_0.5_Mn	A1 + A2	A1 + A2	CoCrFeMnNi_2_V_0.5_	A1	A1
Al_0.3_Cr_2_Fe_0.5_Mn_0.8_	A2	A2	CoCrFeMoNiV_0.5_	IM+	Sigma + A1
Al_0.5_CoCr_0.5_CuMnNi	A1 + A2	**A1**	CoCrFeMoV	IM+	Sigma
Al_0.5_CoCuFeNiV_0.5_	A1	A1	CoCrFeNb_0.5_Ti_0.5_	IM+	Laves
AlCo_0.5_CrCu_0.2_FeMn	B2 + SS	**A2**	CoCrFeNiSi_0.6_	A1	A1
AlCoCrFe	B2 + SS	B2 + A2	CoCr_1.5_Fe_1.5_NiSi_0.2_	A1	A1
AlCoCrFeTi_0.25_	B2 + SS	B2 + A2	CoCuFeMnNiV_0.5_	A1	A1
AlCoCu_0.5_Fe	B2 + SS	B2 + A2	CoFeMnNiTi_0.5_V_0.5_	IM+	**A1**
AlCoCuNiTi_0.25_	B2 + SS	B2 + A1 + A2	CoFeMoNiTi	IM+	Laves
AlCo_2_CrCuNi_3_V	A1	A1	CrCuFeMn	A1 + A2	A1 + A2
AlCrCuFeNiSi_0.25_	B2 + SS	B2 + A2	CrCuFeMnNiTi_0.3_	A1 + A2	A1 + A2
AlCrMoNi_3_W_0.5_	B2 + SS	**A1** + **A2**	CrMoTiV	A2	A2
AlCuFeNi	B2 + SS	B2 + A1 + A2	CrNbNiTiZr	IM+	Laves
Al_2_CoNb_0.2_Ni	B2+SS	**Laves** + **B2** + **A2**	Cr_2_FeNiTi	IM+	Laves+A2
Co_0.2_TaTiV	A2	A2	CuFeMnNiTi_2_	IM+	Laves+A2
CoCr_0.3_Cu_0.2_FeNiV_0.5_	A1	A1	CuFeMnNiV	A1+A2	**Sigma** + **A1**
CoCr_0.5_Fe_2_NiTi_0.25_	A1	A1	Hf_0.5_NbTaW_0.5_Zr	A2	A2
CoCrCu_0.5_FeNi_2_Ti_0.5_V_0.5_	A1	A1	HfNbTaZr	A2	A2

Recall that IM+ phase is the appearance of Sigma or Laves phase together with the potential existence of other phases such as the A1 and A2 solid solution phases. In the real phase column, the detailed phase information is listed. The eight HEAs whose measured phases differ from predictions are underlined. (XRD patterns cannot reveal if the B2 phase exists with or without the A2 phase because of diffraction peaks overlapping. Moreover, in HEAs, B2 usually tends to form with A2. Thus, B2 is listed together with A2 in the real phase information).

## Discussion

For the first time, a method predicting the phase formation of HEAs based solely on the binary phase diagrams is demonstrated and validated. The information on elemental mixing and phase separation from binary phase diagrams has provided success to the phenomenological approach presented. Considering the atomic mobility at high temperatures and presumed pairwise additivity of atomic pair interactions, this information from binary diagrams is used combinatorially to evaluate HEA phases formation. The initial success of using PFP_X_ and PSP, defined using binary phase diagrams, in predicting the corresponding single phase and mixed phase HEAs, prompted us to apply this method to include more phases. The inter-correlated roles of these parameters are noted, and their combined effect must be considered in designing HEAs. We have included in our study the majority of the entire available HEA database, excluding those containing line compounds and the minor phases. Visualisation reveals robust HEA phase formation regions in the parameter space. ML enables the quantification of HEA phase formation, yielding an average single phases prediction success rate of about 90% for the Level 1 and Level 2, and more than 80% for Level 3. The ML success rates obtained from the as-cast HEAs, or the as-cast and annealed HEAs vary marginally. Thus, the model works well for the as-cast and the high temperature annealed HEAs. Considering that these are the most common HEA preparation methods, our model can be applied to most HEA synthesis situations. High accuracy is obtained even with small training set percentages. This implies that the phase formation parameters are well defined and efficient in prediction. Most HEA phase prediction models do not have experiment validation. The high experimental validation success rate of this method is indicative of its reliability. Moreover, ML can predict the phases of the new HEAs to expand the current database and phase parameter regions.

Compared with the other large database statistical approaches, Tancret *et al*. combined Gaussian Process using nine thermodynamic and atomistic parameters with CALPHAD to predict the formation of over 60 single solid solution phase HEAs^25^. The performance of the model has high precision but low recall. Many of the alloys predicted as single solid solution phase HEAs by this method have a high chance of being single solid solution phase HEAs, but many potential single solid solution phase HEAs are misidentified as mixed phases HEAs. Additionally, the exact phase of a HEA such as BCC or IM cannot be predicted. As a comparison, our method has high precision and high recall, and gives specific phase formation information.

Another model by Kube *et al*. assigned values called stabilising abilities (${\beta }_{i}$) to seven specific elements Al, Co, Cr, Cu, Fe, Mn, and Ni representing their strength in stabilising FCC or BCC formation. The ${\beta }_{i}$’s are optimised by ordinal logistic regression based on a database of over 2000 sputter deposited HEAs from a high-throughput experiment^26^. This method is efficient in separating out FCC and BCC single phase HEAs. But mixed FCC and BCC phase cannot be separated from the prior phases. Moreover, other phases such as HCP and IM were not studied. Our method has no element preference and a higher number of phases can be predicted.

Neural network models trained by Huang *et al*.^28^ and Islam *et al*.^27^ based on the thermodynamic and atomistic parameters only predict the phase categories such as the formation of solid solution, IM, or their mixture. Details of phase information are not predicted. Thus far, no published model uses these parameters to predict detailed phase formation accurately. However, with the phenomenological parameters in this article, we have proved theoretically and experimentally the ability to predict detailed phase formation with high accuracy.

To summarise, the advantages of our approach are the following:

1. Indiscriminate HEA selection feasibility: Some prediction methods such as CALPHAD are limited by the availability and depth of proprietary databases. Our method is based solely on binary phase diagrams for which there exist plentiful easily accessible data.
2. Phase region expansion ability: New HEAs are predicted with a high success rate outside the regions where 614 HEA phases are currently known.
3. Parameter-phase relevance: Unlike the traditional thermodynamic parameters, our parameters directly determine the formation of the corresponding phases. Detailed phase formation can be predicted.
4. Ease of computing: Methods such as ab initio molecular dynamics require high computation capability. This model can be run on a laptop, and no high-performance computing facilities are needed.

Looking forward, the model can be enhanced by:

1. Including additional physical parameters into ML: Each parameter represents one factor influencing the phase formation. To fundamentally improve the phase prediction accuracy, ML can be further developed to uncover the hidden phase formation principles. For example, the proper use of mixing enthalpy can influence IM formation in a meaningful way.
2. Interaction with other models: On the one hand, the outcome of the current model can serve as a fast screening for other methods. Our approach can accurately and quickly find the alloy systems with desired phases to serve specific purposes. Beyond this, other methods such as CALPHAD can conduct an in-depth study on these systems for detailed information about the phase transition under different temperatures or the precise control of secondary phase precipitation by fine adjustment of the composition. Certainly, thermodynamic parameters from CALPHAD can help the ML prediction.

## Method

### Melting temperature

The T_m_ is calculated from the liquidus temperatures in binary phase diagrams. c_i_ and c_j_ are the atomic percentages of the elements i and j. For the binary pair i-j, binary liquidus temperatures T_i–j_ can be found at the composition where i and j element relative ratio is c_i_:c_j_. T_m_ of the whole system will be calculated by the following Eq. (1):

1 $${{\rm{T}}}_{{\rm{m}}}=\frac{{\sum }_{{\rm{i}}\ne {\rm{j}}}{{\rm{T}}}_{{\rm{i}}-{\rm{j}}}\times {{\rm{c}}}_{{\rm{i}}}\times {{\rm{c}}}_{{\rm{j}}}}{{\sum }_{{\rm{i}}\ne {\rm{j}}}{{\rm{c}}}_{{\rm{i}}}\times {{\rm{c}}}_{{\rm{j}}}}$$

Where the summation is over all the i-j pairs in the alloy system.

### Methods of calculating parameters

#### Calculating PFP_x_

The method of calculating binary phase field percentage, X_i–j_, uses line segments at T_pf_. X_i–j_ is the percentage of the line segment between the two intersection points of an isotherm at T_pf_ and the phase boundary of phase X. It is assumed that the phases at solidification are directly related to the phases occurring at T_pf_ because the phase transformation occurs for a longer duration near T_pf_ as opposed to near T_m_ due to a decreasing cooling rate when the temperature is decreased.

PFP_x_ is calculated from X_i–j_ by Eq. (2), where c_i_ and c_j_ are the atomic percentages of i-th and j-th elements.

2 $${{\rm{P}}{\rm{F}}{\rm{P}}}_{{\rm{X}}}=\,\frac{{\sum }_{i\ne {\rm{j}}}{{\rm{X}}}_{{\rm{i}}-{\rm{j}}}\times {{\rm{c}}}_{{\rm{i}}}\times {{\rm{c}}}_{{\rm{j}}}}{{\sum }_{i\ne {\rm{j}}}{{\rm{c}}}_{{\rm{i}}}\times {{\rm{c}}}_{{\rm{j}}}}\div100\,{\rm{ \% }}$$

**Figure 5:**
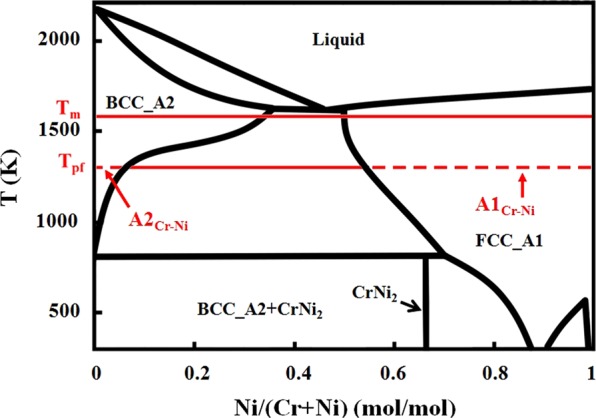
Demonstration of the binary phase field percentage calculation. The binary phase diagram Cr-Ni is used to determine the A1_Cr–Ni_ and A2_Cr–Ni_ for the HEA Al_2_CoCrCuNi.

An example of the PFP_x_ calculation is shown in Fig. 5. Here the Cr-Ni phase diagram is used to calculate A2_Cr–Ni_and A1_Cr–Ni_. Using the HEA Al_2_CoCrCuNi, with a T_m_ = 1569 K, the phases are assumed to form at T_pf_ = 1255 K, and the method gives A2_Cr–Ni_ = 5% and ${\rm{A}}{1}_{{\rm{Cr}}-{\rm{Ni}}}$ = 44%.

### Calculating PSP

The binary phase separation percentage for atomic pair i and j, Separation_i–j_, is calculated using the line segment method at T_pf_. The remaining phase percentage is Mixing_i–j_. The PSP for a HEA is defined by Eq. (3):

3 $$\mathrm{PSP}\,=\,\frac{{\sum }_{{\rm{i}}\ne {\rm{j}}}{{\rm{Separation}}}_{{\rm{i}}-{\rm{j}}}\times {{\rm{c}}}_{{\rm{i}}}\times {{\rm{c}}}_{{\rm{j}}}}{{\sum }_{{\rm{i}}\ne {\rm{j}}}{{\rm{Mixing}}}_{{\rm{i}}-{\rm{j}}}\times {{\rm{c}}}_{{\rm{i}}}\times {{\rm{c}}}_{{\rm{j}}}}$$

**Figure 6:**
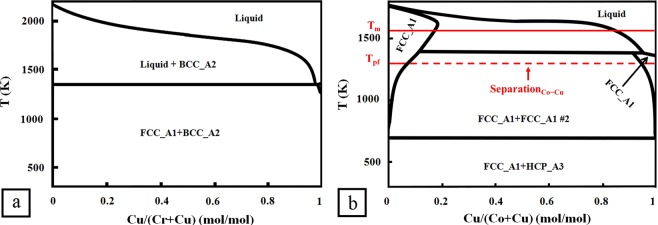
Two binary phase diagrams used to determine binary phase separation percentage for HEA Al_2_CoCrCuNi. (**a**) Phase diagram of Cr-Cu to show a complete phase separation effect. (**b**) Overlay of the Co-Cu phase diagram illustrating the line segment method to determine the Separation_Co–Cu_ for the HEA Al_2_CoCrCuNi.

The atomic pairs with the separation effect are identified on phase diagrams by the presence of two bounding pure solid solution phases with no additional single phase present between the two. For example, a strong phase separation effect exists on the phase diagram of Cr-Cu (Fig. 6a) where Cr and Cu never dissolve into the same phase matrix.

In certain cases, at high temperatures, the mixing entropy term is large enough to overcome the positive mixing enthalpy and results in a negative Gibbs free energy for forming the solid solution. This makes it possible to have the two elements mixed marginally. Co-Cu in Fig. 6b is a typical example where two atoms separate at low temperature and mixing exists at high temperature. The Co-Cu phase diagram is used to calculate the ${{\rm{Separation}}}_{{\rm{Co}}-{\rm{Cu}}}$ by the line segment method. The HEA Al_2_CoCrCuNi is used again. This method gives a Separation_Co–Cu_ = 92% and Mixing_Co–Cu_ = 8%. Separation_i–j_ = 0% if the phase separation is absent from a phase diagram.

### Phase formation temperature

For an as-cast HEA, the phase transformation evolves at various temperatures above T_pf_ as it cools from the molten state. The values of PFP_X_ and PSP are different when calculated using different T_pf_. Thus they result in different ML accuracies. To optimise the value of T_pf_, the parameter calculation and corresponding ML were conducted with T_pf_ = 0.7, 0.75, 0.8, 0.85, and 0.9 T_m_. Highest ML accuracy was obtained when T_pf_ = 0.8 T_m_. Of note, the optimised T_pf_ is close to the undercooling temperature.

For the high temperature annealed HEAs, the phases formed during annealing at these high temperatures are locked in during rapid quenching. Thus, T_pf_ is the annealing temperature and the phase formation tendency is determined from the line segment percentages of the binary phase fields present.

### Machine learning

ML was conducted using the data mining software WEKA 3.8^32^. We use Random Forest^33^ with 300 trees to perform this classification task. The features are the parameters defined for the three levels of the database partition. Each database level is divided randomly into training and test sets. The ML algorithm establishes and optimises decision trees based on the training set. These trees are used to predict the phases of HEAs in the test set based on their features. The performance of the ML model is accessed by 2, 3, 4, 5, and 10-fold cross-validations, which, in Table 1, correspond to training set percentages of 50%, 67%, 75%, 80%, and 90%. An F1 score, as a weighted average of precision and recall model evaluation metrics, is used to denote the success rate of prediction. Each cross-validation is conducted for 20 times and then the average F1 score is obtained. After the optimisation, new HEAs are predicted.

### Alloy validation experiment

The 42 predicted HEAs used to validate our model were all prepared by suction casting. These HEAs are created by first making master ingots. These ingots are made from elements with a minimum purity of 99.7 wt%. The elements are arc-melted in a water-cooled copper hearth in a high purity argon atmosphere and are melted three times to ensure homogeneous mixing. The ingots are then suction-casted into a copper mould making 3 mm diameter rods. Structure investigations are carried out with XRD analysis using a Cu Kα radiation on a PANalytical Empyrean diffractometer.

### Database description

679 HEAs have been collected from literature in the supplementary material. Structural data used in our model is predominantly from XRD measurements. When transmission electron microscopy (TEM) data is available and it can reveal the hidden patterns from XRD results, the higher resolution TEM data will supersede the XRD data. The study is limited to 614 HEAs formed in the as-cast state or those annealed at temperatures higher than 0.7 T_m_. Most of the heat-treated HEAs were annealed above 0.7 T_m_. The high-temperature annealed HEAs are included since the formation entropy can contribute more Gibbs free energy change at the higher temperatures. Mechanically alloyed HEAs are not included because ball milling tends to retain metastable phases.

## Supplementary information

Supplementary Information

## Data Availability

The authors confirm that the main data used in this study are available within the supplementary materials
