# Publisher Correction: High Entropy Alloys Mined From Binary Phase Diagrams

**DOI:** 10.1038/s41598-020-58758-1

**Published:** 2020-01-30

**Authors:** Jie Qi, Andrew M. Cheung, S. Joseph Poon

**Affiliations:** Department of Physics, University of Virginia, Charlottesville, VA 22904-4714 USA

Correction to: *Scientific Reports* 10.1038/s41598-019-50015-4, published online 29 October 2019

This Article contains errors in Table 1. In the HTML and PDF versions of this Article, there are values missing in the first row of Training Set Percentage (Level 1). The correct Table 1 appears below.

**Table 1:** ML results and count of HEAs for the three levels of the study. ML prediction success rates for the as-cast HEAs and the as-cast + annealed HEAs in different phases are listed. The success rates are F1 scores. Counts of HEAs and phases for the as-cast HEAs and the as-cast + annealed HEAs in different phases are listed.

ML Prediction Success Rate (%)								
		As-Cast [As-Cast + Annealed]						
Level	Phase Category	Overall	A1	A2	A1-A2	A3	B2+SS	IM+
	Training Set Percentage (%)							
Level 1	90	90 [89]	89 [91]	95 [93]	81 [76]	100 [100]		
	80	89 [89]	88 [91]	95 [94]	80 [76]	100 [98]		
	75	90 [88]	89 [90]	95 [93]	81 [73]	100 [100]		
	67	89 [87]	87 [89]	95 [93]	79 [73]	100 [98]		
	50	89 [87]	87 [89]	95 [93]	80 [74]	98 [98]		
Level 2	90	85 [85]	84 [87]	94 [94]	69 [62]	100 [100]	86 [87]	
	80	84 [85]	83 [87]	94 [93]	67 [62]	100 [98]	85 [86]	
	75	85 [84]	84 [86]	94 [93]	68 [60]	100 [98]	86 [85]	
	67	85 [83]	83 [85]	94 [92]	68 [59]	100 [96]	85 [85]	
	50	83 [84]	79 [85]	94 [92]	64 [61]	100 [95]	84 [85]	
Level 3	90	82 [80]	81 [81]	92 [87]	64 [61]	100 [100]	84 [85]	78 [77]
	80	81 [79]	80 [80]	91 [87]	63 [58]	100 [100]	84 [83]	78 [76]
	75	81 [79]	80 [80]	91 [87]	63 [59]	100 [100]	84 [83]	76 [74]
	67	80 [78]	80 [80]	91 [85]	63 [60]	100 [100]	83 [82]	74 [73]
	50	78 [77]	78 [77]	90 [86]	62 [56]	100 [98]	80 [81]	73 [71]
**Total Alloy Counts**								
		**As-Cast [As-Cast + Annealed]**						
**Phase Category**		**Overall**	**A1**	**A2**	**A1-A2**	**A3**	**B2+SS**	**IM+**
**Level**								
Level 1		235 [317]	69 [118]	101 [121]	61 [72]	4 [6]		
Level 2		375 [486]	69 [118]	101 [121]	61 [72]	4 [6]	140 [169]	
Level 3		470 [614]	69 [118]	101 [121]	61 [72]	4 [6]	140 [169]	95 [128]

