# Supplementary material for: High Entropy Alloys Mined From Binary Phase Diagrams
Source: Sci Rep. 2019 Oct 29;9:15501. doi: 10.1038/s41598-019-50015-4 (PMC6820750; doi:10.1038/s41598-019-50015-4)
Supplement: Supplementary file 1 — Supplementary Information [file 41598_2019_50015_MOESM1_ESM.pdf]

Supplementary Material

Jie Qi, Andrew M. Cheung, and S. Joseph Poon, High Entropy Alloys Mined From Binary Phase Diagrams, 2019.

Section 1: HEA database:

The list of HEAs with information about compositions, alloy preparation methods, and phases is presented in Table. S1. The database includes HEAs up to early 2019, containing 193 alloy systems, 679 compositions, and 1084 reports. A report represents an alloy being prepared with a specific method in a specific paper. Some alloys have multiple reports because they are mentioned in different papers or prepared with different methods. The database is from augmenting the database collected by Miracle, D. B. & Senkov, O. N. in 2016,[1] in which 112 alloy systems, 408 compositions, and 648 reports are included. Many notations from their work are being used here.

The database is sorted alphabetically. The subscripts in each composition represent the atomic ratios of different elements. If a subscript is absent for an element, the atomic ratio is 1. For example: $AlCoCrCu_{0.5}Fe$ represents an alloy with Al, Co, Cr Cu, and Fe with relative atomic ratios to be 1:1:1:0.5:1, or with atomic percentages to be 22%, 22%, 22%, 11%, and 22% respectively.

The first column is the alloy system, with only the element information listed.

The second column is the detail composition of HEAs.

The third column is the preparation methods. AC=As-cast; MA=Mechanical alloying; SPS=Spark Plasma Sintered; LC=Laser coating/cladding; MS =Melt spun; SP =Sputtering; SQ=Splat quenched; AM=Additive manufacturing; FC=Furnace cooled; FOR=Forged; WQ=Water quenched; HP=Hot pressing; HPT=High Pressure Torsion; CR=Cold rolled; HR=Hot rolled. For cold/hot roll, the thickness reduction is included in the parenthesis. For the annealing process, the annealing temperature and time are listed. The temperature is recorded with the unit either in Kelvin (K) or Celsius degree (C). The time is recorded with the unit in minutes (min), hour (h), or days. The pressure is recorded with the unit in Megapascal (MPa).

The fourth column is the phase category. SS=disordered solid solution phase; IM=Intermetallic; AM= Amorphous; Unk=Unknown.

The fifth column is the detailed phase information. Notations FCC, BCC, HCP, and σ correspond to phases with Strukturbericht designations A1, A2, A3, and $D8_b$. Laves represents phases C14, C15, and C36. All the other notations are in Strukturbericht designations. IM is the phase which shows several peaks on X-Ray Diffraction (XRD) patterns but without any other information. Unk is unknown XRD peak and phase.

The sixth column is the reference for each HEA report.

Table S1: HEA database up to early 2019.

**Alloy System**	**Composition**	**Preparation Methods**	**Phase Category**	**Phases**	**Ref**
AgAlCoCrCuNi	AgAlCoCrCuNi	AC	SS+IM	2FCC+B2	1
AgCoCrFeMnNi	AgCoCrFeMnNi	AC	SS	2FCC	2
AlAuCoCrCuNi	AlAuCoCrCuNi	AC	SS+IM	FCC+$L1_0$	1
AlBCoCrCuFeNi	$Al_{0.5}B_{0.2}CoCrCuFeNi$	AC	SS+IM	FCC+IM	1
	$Al_{0.5}B_{0.6}CoCrCuFeNi$	AC	SS+IM	FCC+IM	1
	$Al_{0.5}BCoCrCuFeNi$	AC	IM	$L1_2$+IM	1
AlCCoCrFeNi	$AlC_{0.1}CoCrFeNi$	AC	SS+IM	BCC+IM	1
	$AlC_{0.2}CoCrFeNi$	AC	SS+IM	BCC+IM	1
	$AlC_{0.3}CoCrFeNi$	AC	SS+IM	BCC+IM	1
	$AlC_{0.4}CoCrFeNi$	AC	SS+IM	BCC+IM	1
	$AlC_{0.5}CoCrFeNi$	AC	SS+IM	BCC+IM	1
	AlCCoCrFeNi	AC	SS+IM	BCC+A9+IM	1
	$AlC_{1.5}CoCrFeNi$	AC	SS+IM	BCC+A9+IM	1
AlCoCrCuFe	$AlCoCrCu_{0.5}Fe$	AC	SS+IM	FCC+B2	1
	AlCoCrCuFe	MA+SPS	SS+IM	FCC+B2+σ	1
		MA	SS	FCC+BCC	1
AlCoCrCuFeMnNi	AlCoCrCuFeMnNi	AC	SS+IM	FCC+2B2	1
AlCoCrCuFeMnNiTiV	$Al_{0.125}CoCrCuFeMnNiTiV$	AC	SS	BCC+FCC	1
	$Al_{0.25}CoCrCuFeMnNiTiV$	AC	SS	BCC	1
	$Al_{0.67}CoCrCuFeMnNiTiV$	AC	SS+IM	BCC+$D0_{22}$+IM	1
AlCoCrCuFeMoNi	$AlCoCrCuFeMo_{0.2}Ni$	AC	SS	BCC+FCC	1
	$AlCoCrCuFeMo_{0.4}Ni$	AC	Unk	BCC+Unk	1
	$AlCoCrCuFeMo_{0.6}Ni$	AC	Unk	BCC+Unk	1
	$AlCoCrCuFeMo_{0.8}Ni$	AC	Unk	BCC+Unk	1
	AlCoCrCuFeMoNi	AC	Unk	BCC+Unk	1
AlCoCrCuFeNi	$Al_{0.2}CoCrCu_{0.2}FeNi_2$	AC+1200C/24h+CR+700C/20h	SS+IM	FCC+ $L1_2$	3
	$Al_{0.2}CoCrCu_{0.2}FeNi_2$	AC+1200C/24h+CR+800C/1h	SS	FCC	3
	$Al_{0.25}CoCrCu_{0.75}FeNi$	AC	SS	FCC	1
	$Al_{0.3}CoCrCu_{0.3}FeNi$	AC+1100C/6h+WQ/FQ	SS	FCC	4
	$Al_{0.3}CoCrCu_{0.5}FeNi$	AC+1100C/24h/FC	SS+IM	FCC+$L1_2$	1
	$Al_{0.3}CoCrCuFeNi$	AC	SS	FCC	1
		AC	SS	FCC	1
	$Al_{0.5}CoCrCu_{0.5}FeNi$	AC	SS	2FCC	1

		AC	SS	FCC	1
		AC	SS	FCC	5
	$Al_{0.5}CoCrCu_{0.5}FeNi_2$	AC	SS+IM	$FCC+L1_2$	1
	$Al_{0.5}CoCrCu_{0.5}Fe_2Ni$	AC	SS	FCC	5
	$Al_{0.5}CoCrCu_{0.5}Fe_3Ni$	AC	SS	FCC	5
	$Al_{0.5}CoCrCu_{0.5}Fe_{3.5}Ni$	AC	SS	FCC	5
	$Al_{0.5}CoCrCuFeNi$	AC	SS	FCC	1
		AC	SS	FCC	1
		AC	SS	FCC	1
		AC	SS	2FCC	1
		AC	SS	FCC	1
		AC	SS	2FCC	1
		AC	SS	2FCC	1
		AC	SS	FCC	1
		AC	SS	FCC	1
		AC+FOR(40%)	SS	BCC+2FCC	1
		AC+FOR(40%)+1100C/24/WQ	SS+IM	2FCC	1
		AC+FOR(40%)+1100C/24/FC	SS	BCC+2FCC	1
		AC+1000C/6h/WQ	SS	2FCC	1
		AC+1000C/6h+CR(80%)+900C/5h	SS	2FCC	1
		AC+1000C/6h+CR(80%)+700C/10h	SS	BCC+2FCC	1
		AC+1000C/6h+CR(80%)+600C/10h	SS	BCC+2FCC	1
		AC+1000C/6h+CR(80%)+500C/10h	SS	BCC+FCC	1
		AC+CR (67%)	SS+IM	BCC+2FCC+B2	1
		AC+CR (67%) +700C/480h	SS+IM	BCC+2FCC+B2+σ	1
		AC+CR (67%) +900C/480h	SS+IM	BCC+2FCC+B2	1
		AC+CR(67%) +1100C/480h	SS+IM	$2FCC+L1_2$	1
		AC+1000C/6h/WQ+CR (84%)	SS+IM	$FCC+L1_2$	1
		AC	SS	2FCC	1
		AC+600C/24h	SS	BCC+FCC	1
	$Al_{0.5}CoCr_3Cu_{0.5}FeNi$	AC	SS	BCC+FCC	5
	$Al_{0.75}CoCrCu_{0.25}FeNi$	AC	SS	BCC+FCC	1
	$Al_{0.8}CoCrCuFeNi$	AC	SS	BCC+2FCC	1
		AC	SS	BCC+FCC	1
		AC	SS	BCC+FCC	1

	$AlCo_{0.5}CrCu_{0.5}FeNi$	AC	IM	B2	1
	$AlCo_{0.5}CrCuFeNi$	AC	SS+IM	BCC+FCC+B2	1
	$AlCoCr_{0.5}Cu_{0.5}FeNi$	AC	SS+IM	BCC+FCC+B2	1
	$AlCoCr_{0.5}CuFeNi$	AC	SS+IM	BCC+FCC+B2	1
	$AlCoCrCu_{0.25}FeNi$	AC	SS	BCC	1
	$AlCoCrCu_{0.5}Fe_{0.5}Ni$	AC	SS	BCC+FCC	1
		AC	SS+IM	FCC+B2	1
	$AlCoCrCu_{0.5}FeNi_{0.5}$	AC	SS+IM	FCC+BCC+B2	5
	$AlCoCrCu_{0.5}FeNi$	AC	SS+IM	FCC+BCC+B2	1
		AC	SS+IM	FCC+B2	1
	$AlCoCrCu_{0.5}FeNi_{1.5}$	AC	SS	BCC+FCC	1
	$AlCoCrCu_{0.5}FeNi_2$	AC	SS	BCC+FCC	1
	$AlCoCrCu_{0.5}FeNi_{2.5}$	AC	SS+IM	BCC+$L1_2$	1
	$AlCoCrCu_{0.5}FeNi_3$	AC	SS+IM	FCC+$L1_2$	1
	$AlCoCrCu_{0.5}Fe_{1.5}Ni$	AC	SS+IM	FCC+B2	1
	$AlCoCrCu_{0.5}Fe_2Ni$	AC	SS+IM	FCC+B2	1
	$AlCoCrCuFe_{0.5}Ni$	AC	SS+IM	BCC+FCC+B2	1
	$AlCoCrCuFeNi_{0.5}$	AC	SS+IM	BCC+FCC+B2	1
	AlCoCrCuFeNi	AC	SS+IM	FCC+B2	1
		AC	SS+IM	BCC+FCC+B2	1
		AC	SS	BCC+FCC	1
		AC	SS	BCC+FCC	1
		AC	SS	BCC+FCC	1
		AC	SS+IM	BCC+FCC+B2	1
		AC	SS	BCC+FCC	1
		AC	SS	BCC+FCC	1
		AC	SS	BCC+FCC	1
		AC+500C/5h	SS	BCC+FCC	1
		AC+600C/5h	SS	BCC+FCC	1
		AC+645C/5h	SS	BCC+FCC	1
		AC+700C/5h	SS	BCC+FCC	1
		AC+800C/5h	SS	BCC+FCC	1
		AC+900C/5h	SS	BCC+FCC	1
		AC+1000C/5h	SS	BCC+FCC	1
		AC	SS	BCC+FCC	1
		AC	SS+IM	FCC+B2	1
		AC+1000C/2h	SS+IM	2FCC+B2	1
		SQ	IM	B2	1
		AC	SS+IM	BCC+2B2+2$L1_2$	1
		AC	SS+IM	FCC+B2	1
		AC	SS	BCC+2FCC	1

		AC+960C/50h+FOR/950C (1000%)	SS+IM	BCC+2FCC+σ	1
		AC	SS	BCC+FCC	1
	$AlCoCrCu_{0.5}FeNi$	AC	SS+IM	BCC+FCC+B2	1
	$AlCoCr_{1.5}Cu_{0.5}FeNi$	AC	SS	BCC	1
	$AlCoCr_2Cu_{0.5}FeNi$	AC	SS+IM	BCC+B2	1
	$AlCo_{1.5}CrCu_{0.5}FeNi$	AC	SS+IM	FCC+B2	1
	$AlCo_2CrCu_{0.5}FeNi$	AC	SS	BCC+FCC	1
	$AlCo_3CrCu_{0.5}FeNi$	AC	SS	BCC+FCC	1
	$AlCo_{3.5}CrCu_{0.5}FeNi$	AC	SS	FCC	1
	$Al_{1.3}CoCrCuFeNi$	AC	SS+IM	FCC+B2	1
	$Al_{1.4}Co_{0.9}Cr_{1.4}Cu_{0.5}Fe_{0.9}Ni$	AC	SS+IM	BCC+B2+Unk	1
	$Al_{1.5}CoCrCu_{0.5}FeNi$	AC	IM	BCC+B2	1
	$Al_{1.5}CoCrCuFeNi$	AC	SS+IM	FCC+B2	1
	$Al_{1.8}CoCrCuFeNi$	AC	SS+IM	FCC+B2	1
	$Al_2CoCrCu_{0.5}FeNi$	AC	IM+SS	B2 +BCC	1
	$Al_2CoCrCuFeNi$	AC	SS+IM	B2+FCC	1
		AC	SS+IM	FCC+B2	1
	$Al_{2.3}CoCrCuFeNi$	AC	SS+IM	FCC+B2	1
	$Al_{2.5}CoCrCuFeNi$	AC	SS+IM	FCC+B2	1
	$Al_{2.8}CoCrCuFeNi$	AC	IM	B2	1
	$Al_3CoCrCuFeNi$	AC	IM	B2	1
	$Al_8Co_{17}Cr_{17}Cu_8Fe_{17}Ni_{33}$	AC	IM+SS	$FCC+L1_2$	6
	$Al_{23}Co_{15}Cr_{23}Cu_8Fe_{15}Ni_{16}$	AC	IM+SS	B2+BCC+FCC	6
AlCoCrCuFeNiSi	$Al_{0.5}CoCrCuFeNiSi_{0.8}$	AC	SS+IM	FCC+BCC+B2+IM	7
	$Al_{0.5}CoCrCuFeNiSi$	AC	SS+IM	FCC+BCC+B2+IM	7
	$Al_{0.5}CoCrCuFeNiSi_{1.2}$	AC	SS+IM	FCC+BCC+B2+IM	7
	$Al_{0.5}CoCrCuFeNiSi_{1.4}$	AC	SS+IM	FCC+BCC+B2+IM	7
	$Al_{0.5}CoCrCuFeNiSi_{1.6}$	AC	SS+IM	FCC+BCC+B2+IM	7
	$Al_{0.5}CoCrCuFeNiSi_{1.8}$	AC	SS+IM	FCC+BCC+B2+IM	7
	$Al_{0.5}CoCrCuFeNiSi_2$	AC	SS+IM	FCC+BCC+B2+IM	7
	$AlCoCrCu_{0.5}FeNiSi$	AC	AM	AM	1
	AlCoCrCuFeNiSi	AC	SS	BCC+FCC	1
AlCoCrCuFeNiTi	$Al_{0.25}CoCrCu_{0.75}FeNiTi_{0.5}$	AC	SS	2FCC	1
	$Al_{0.5}CoCrCu_{0.5}FeNiTi_{0.5}$	AC	SS	2BCC+FCC	1
	$Al_{0.5}CoCrCuFeNiTi_{0.2}$	AC	SS	FCC	1
	$Al_{0.5}CoCrCuFeNiTi_{0.4}$	AC	SS	2BCC+FCC	1
	$Al_{0.5}CoCrCuFeNiTi_{0.6}$	AC	SS	2BCC+FCC	1
	$Al_{0.5}CoCrCuFeNiTi_{0.8}$	AC	SS+IM	2BCC+FCC+σ	1
	$Al_{0.5}CoCrCuFeNiTi$	AC	SS+IM	2BCC+FCC+σ	1
	$Al_{0.5}CoCrCuFeNiTi_{1.2}$	AC	SS+IM	$2BCC+FCC+NiTi_2+σ$	1
	$Al_{0.5}CoCrCuFeNiTi_{1.4}$	AC	SS+IM	$BCC+FCC+B2+NiTi_2$	1

	$Al_{0.5}CoCrCuFeNiTi_{1.6}$	AC	SS+IM	BCC+FCC+B2+$NiTi_2$	1
	$Al_{0.5}CoCrCuFeNiTi_{1.8}$	AC	SS+IM	BCC+FCC+B2+$NiTi_2$	1
	$Al_{0.5}CoCrCuFeNiTi_2$	AC	SS+IM	BCC+FCC+B2+$NiTi_2$	1
	$Al_{0.75}CoCrCu_{0.25}FeNiTi_{0.5}$	AC	SS	2BCC	1
	$AlCoCrCu_{0.25}FeNiTi_{0.5}$	AC	SS	2BCC	1
	$AlCoCrCu_{0.5}FeNiTi_{0.5}$	AC	SS	2BCC	1
	AlCoCrCuFeNiTi	AC	SS+IM	BCC+FCC+B2	1
AlCoCrCuFeNiTiV	AlCoCrCuFeNiTiV	AC	SS	BCC+FCC	1
		SQ	SS	BCC	1
AlCoCrCuFeNiV	$Al_{0.5}CoCrCuFeNiV_{0.2}$	AC	SS	2FCC	1
	$Al_{0.5}CoCrCuFeNiV_{0.4}$	AC	SS	BCC+FCC	1
	$Al_{0.5}CoCrCuFeNiV_{0.6}$	AC	SS+IM	BCC+FCC+σ	1
	$Al_{0.5}CoCrCuFeNiV_{0.8}$	AC	SS+IM	BCC+FCC+σ	1
	$Al_{0.5}CoCrCuFeNiV$	AC	SS+IM	BCC+FCC+σ	1
	$Al_{0.5}CoCrCuFeNiV_{1.2}$	AC	SS	BCC+FCC	1
	$Al_{0.5}CoCrCuFeNiV_{1.4}$	AC	SS	BCC+FCC	1
	$Al_{0.5}CoCrCuFeNiV_{1.6}$	AC	SS	BCC+FCC	1
	$Al_{0.5}CoCrCuFeNiV_{1.8}$	AC	SS	BCC+FCC	1
	$Al_{0.5}CoCrCuFeNiV_2$	AC	SS	BCC+FCC	1
	$AlCoCrCuFeNiV_{0.2}$	MA+LC	SS	FCC	8
	$AlCoCrCuFeNiV_{0.5}$	MA+LC	SS	FCC+BCC	8
	$AlCoCrCuFeNiV_{0.8}$	MA+LC	SS	FCC+BCC	8
	AlCoCrCuFeNiV	AC	SS	BCC+FCC	1
		MA+LC	SS	BCC+FCC	8
AlCoCrCuFeSiTi	AlCoCrCuFeSiTi	AC	SS	BCC+FCC	9
AlCoCrCuNi	$AlCoCrCu_{0.5}Ni$	AC	SS+IM	BCC+FCC+B2	1
		AC	SS+IM	BCC+FCC+B2	1
		SP	SS	BCC	1
	AlCoCrCuNi	AC	SS	BCC+FCC	1
		AC	SS+IM	BCC+FCC+B2	1
AlCoCrCuNiTi	AlCoCrCuNiTi	AC	SS	2BCC+FCC	1
AlCoCrCuNiTiY	$AlCoCrCuNiTiY_{0.5}$	AC	SS+IM	BCC+FCC+C15+$L2_1$	1
	$AlCoCrCuNiTiY_{0.8}$	AC	SS+IM	BCC+C15+$L2_1$	1
	AlCoCrCuNiTiY	AC	SS+IM	BCC+C15+$L2_1$+Unk	1
AlCoCrFeMnNi	$Al_{0.1}CoCrFeMnNi$	AC	SS	FCC	1
	$Al_{0.15}CoCrFeMnNi$	AC	SS	FCC	1
	$Al_{0.2}CoCrFeMnNi$	AC	SS	FCC	1
	$Al_{0.38}CoCrFeMnNi$	AC	SS	FCC	1
	$Al_{0.43}CoCrFeMnNi$	AC	SS	FCC	1
	$Al_{0.5}CoCrFeMnNi$	AC	SS+IM	BCC+FCC+B2	1
	$Al_{0.56}CoCrFeMnNi$	AC	SS+IM	BCC+FCC+B2	1
	$Al_{0.62}CoCrFeMnNi$	AC	SS+IM	BCC+FCC+B2	1

	$Al_{0.68}CoCrFeMnNi$	AC	SS+IM	BCC+FCC+B2	1
	$Al_{0.75}CoCrFeMnNi$	AC	SS+IM	BCC+FCC+B2	1
	$Al_{0.81}CoCrFeMnNi$	AC	SS+IM	BCC+FCC+B2	1
	$Al_{0.88}CoCrFeMnNi$	AC	SS+IM	BCC+FCC+B2	1
	$Al_{0.95}CoCrFeMnNi$	AC	SS+IM	BCC+B2	1
	Al1.25CoCrFeMnNi	AC	SS+IM	BCC+B2	1
AlCoCrFeMnNiV	$Al_{0.4}CoCrFeMnNiV$	AC+700C/20h	IM	σ	1
	$AlCo_{0.5}Cr_{0.5}Fe_{0.5}MnNiV_{0.5}$	AC+700C/20h	SS	BCC	1
	$AlCo_{0.5}Cr_{0.5}Fe_{0.5}MnNiV$	AC+700C/20h	SS	BCC	1
	$AlCo_{1.5}Cr_2Fe_{1.5}Mn_2NiV$	AC+700C/20h	IM	σ	1
AlCoCrFeMo	$AlCo0.5CrFeMo_{0.5}$	AC	SS+IM	BCC+B2+σ	10
	$AlCoCrFeMo_{0.5}$	AC	SS+IM	BCC+B2+σ	10
	$AlCo_{1.5}CrFeMo_{0.5}$	AC	SS+IM	BCC+B2+σ	10
	$AlCo_2CrFeMo_{0.5}$	AC	SS+IM	BCC+FCC+σ	10
	$AlCoCrFeMo_{0.5}$	AC	IM	B2+σ	11
AlCoCrFeMoNi	$Al_{0.3}CoCrFeMo_{0.1}Ni$	AC	SS	FCC	1
		AC	SS	FCC	1
		AC+700C/144h/WQ	SS+IM	FCC+B2+σ	1
	$AlCo_{0.5}CrFeMo_{0.5}Ni$	AC	SS+IM	BCC+B2+σ	1
	$AlCoCr_{0.5}FeMo_{0.5}Ni$	AC	IM	B2+σ	1
	$AlCoCrFe_{0.6}Mo_{0.5}Ni$	AC	IM	B2+σ	1
	$AlCoCrFeMo_{0.1}Ni$	AC	SS	BCC	1
	$AlCoCrFeMo_{0.2}Ni$	AC	SS+IM	BCC+IM	1
	$AlCoCrFeMo_{0.3}Ni$	AC	SS+IM	BCC+IM	1
	$AlCoCrFeMo_{0.4}Ni$	AC	SS+IM	BCC+IM	1
	$AlCoCrFeMo_{0.5}Ni_{0.5}$	AC	IM	B2+σ	11
	$AlCoCrFeMo_{0.5}Ni$	AC	SS+IM	BCC+IM	1
		AC	SS+IM	BCC+σ	1
		AC	SS+IM	BCC+σ	1
		AC	IM	B2+σ	11
	$AlCoCrFeMo_{0.5}Ni_{1.5}$	AC	SS+IM	FCC+B2+σ	11
	$AlCoCrFeMo_{0.5}Ni_2$	AC	SS+IM	FCC+B2+σ	11
	$AlCoCrFe_{1.5}Mo_{0.5}Ni$	AC	SS+IM	BCC+σ	1
	$AlCoCrFe_2Mo_{0.5}Ni$	AC	SS+IM	BCC+σ	1
	$AlCoCr_{1.5}FeMo_{0.5}Ni$	AC	IM	B2+σ	1
	$AlCoCr_2FeMo_{0.5}Ni$	AC	IM	B2+σ	1
	$AlCo_{1.5}CrFeMo_{0.5}Ni$	AC	SS+IM	BCC+B2+σ	1
	$AlCo_2CrFeMo_{0.5}Ni$	AC	SS+IM	BCC+FCC+σ	1
AlCoCrFeNbNi	$AlCoCrFeNb_{0.1}Ni$	AC	SS+IM	BCC+C14	1
		AC	SS	BCC	1
	$AlCoCrFeNb_{0.25}Ni$	AC	SS+IM	BCC+C14	1
		AC	SS+IM	BCC+C14	1

	$AlCoCrFeNb_{0.5}Ni$	AC	SS+IM	BCC+C14	1
		AC	SS+IM	BCC+C14	1
	$AlCoCrFeNb_{0.75}Ni$	AC	SS+IM	BCC+C14	1
		AC	SS+IM	BCC+C14	1
AlCoCrFeNi	$Al_{0.1}CoCrFeNi$	AC	SS	FCC	1
	$Al_{0.25}CoCrFeNi$	AC	SS	FCC	1
		AC+1100C/24h/WQ	SS	FCC	1
		AC+1100C/24h/WQ+CR(50%)	SS	FCC	1
		AC+1100C/24h/WQ	SS	FCC	1
	$Al_{0.3}CoCrFeNi$	AC	SS+IM	$FCC+L1_2$	1
		AC+700C/72h/WQ	SS+IM	$FCC+L1_2$	1
		AC+900C/72h/WQ	SS+IM	FCC+B2	1
		AC	SS+IM	$FCC+L1_2$	1
		AC	SS	FCC	1
		AC+700C/144h/WQ	SS+IM	FCC+B2	1
		AC	SS	FCC	1
		AC+700C/20h	SS	FCC	1
		AC	SS+IM	$FCC+L1_2$	12
		AC+1100C/48h+CR(90%)+1100C/1h	SS	FCC	13
		AC+1100C/48h+CR(90%)+1000C/1h	SS+IM	FCC+B2	13
		AC+1100C/48h+CR(90%)+900C/1h	SS+IM	FCC+B2	13
		AC+1100C/48h+CR(90%)+800C/1h	SS+IM	FCC+B2	13
		AC+CR(90%)+1423K/1h	SS	FCC	14
		AC+CR(90%)+1423K/1h+973K/50h	SS+IM	FCC+B2	14
		AC+CR(90%)+1423K/1h+823K/150h	SS+IM	$FCC+ L1_2$	14
		AC+CR(90%)+1423K/2min	SS	FCC	14
		AC+CR(90%)+1423K/2min+923/50h	SS+IM	$FCC+ L1_2+B2$	14
	$Al_{0.3}CoCr_2FeNi$	AC+700C/20h	SS+IM	BCC+FCC+σ	1
	$Al_{0.348}CoCrFeNi$	AC	SS+IM	FCC+BCC+B2	15
		AC+1050C/120h	SS+IM	FCC+B2	15
	$Al_{0.375}CoCrFeNi$	AC	SS	FCC	1
		AC+1100C/24h/WQ	SS	FCC	1
		AC+1100C/24h/WQ+CR(50%)	SS	FCC	1
		AC+1100C/24h/WQ	SS	FCC	1

	$Al_{0.4}CoCrFeNi$	AC	SS	FCC	1
	$Al_{0.41}CoCrFeNi$	AC	SS	FCC	16
	$Al_{0.44}CoCrFeNi$	AC	SS+IM	FCC+B2+Unk	1
	$Al_{0.5}CoCrFeNi$	AC	SS	BCC+FCC	1
		AC	SS	BCC+FCC	1
		AC+1100C/24h/WQ	SS	BCC+FCC	1
		AC+1100C/24h/WQ+CR(50%)	SS	BCC+FCC	1
		AC+1100C/24h/WQ	SS	BCC+FCC	1
		AC	SS	BCC+FCC	1
		AC+350C/24h/WQ	SS	2BCC+FCC	1
		AC+500C/24h/WQ	SS	2BCC+FCC	1
		AC+650C/24h/WQ	SS	2BCC+FCC	1
		AC+800C/24h/WQ	SS	2BCC+FCC	1
		AC+950C/24h/WQ	SS	2BCC+FCC	1
		AC	SS	BCC+FCC	1
	$Al_{0.57}CoCrFeNi$	AC	SS+IM	BCC+FCC+B2	16
	$Al_{0.6}CoCrFeNi$	AC	SS+IM	BCC+FCC+B2	12
	$Al_{0.7}CoCrFeNi$	AC	SS+IM	BCC+FCC+B2	1
		AC	SS+IM	FCC+BCC+B2	15
		AC+1050C/120h	SS+IM	FCC+B2	15
	$Al_{0.7}CoCrFe_2Ni$	AC	SS+IM	FCC+BCC+B2	17
	$Al_{0.7}CoCr_{1.5}Fe_{1.5}Ni$	AC	SS+IM	BCC+B2	18
		AC + 673K/2h	SS+IM	BCC+B2	18
		AC + 773K/2h	SS+IM	BCC+B2	18
		AC + 823K/2h	SS+IM	BCC+B2 +σ	18
		AC + 873K/2h	SS+IM	BCC+B2 +σ	18
		AC + 973K/2h	SS+IM	BCC+FCC+B2 +σ	18
		AC + 1073K/2h	SS+IM	BCC+FCC+B2 +σ	18
		AC + 1173K/2h	SS+IM	BCC+FCC+B2	18
		AC + 1273K/2h	SS+IM	BCC+FCC+B2	18
	$Al_{0.74}CoCrFeNi$	AC	SS+IM	FCC+BCC+B2	16
	$Al_{0.75}CoCrFeNi$	AC+1100C/24h/WQ	SS+IM	FCC+BCC	1
		AC	SS	BCC+FCC	1
		AC+1100C/24h/WQ	SS	BCC+FCC	1
		AC+1100C/24h/WQ+CR(50%)	SS	BCC+FCC	1
	$Al_{0.8}CoCrFeNi$	AC	SS+IM	BCC+FCC+B2	1
	$Al_{0.85}CoCrFeNi$	AC	SS+IM	BCC+B2	12
	$Al_{0.875}CoCrFeNi$	AC	SS	BCC	1
		AC+1100C/24h/WQ	SS+IM	FCC+B2	1

		AC+1100C/24h/WQ+CR(50%)	SS+IM	FCC+B2	1
		AC+1100C/24h/WQ	SS	BCC+FCC	1
	$Al_{0.9}CoCrFeNi$	AC	SS+IM	BCC+B2	1
	$Al_{0.92}CoCrFeNi$	AC	SS+IM	BCC+B2	16
	AlCoCrFeNi	AC	IM	B2	1
		AC	SS+IM	BCC+B2	1
		AC	IM	$B2+L1_2$	1
		AC	IM	B2	1
		AC	IM	B2	1
		AC+1100C/24h/WQ	SS+IM	FCC+B2	1
		AC	SS	BCC	1
		AC+1100C/24h/WQ	SS+IM	FCC+BCC	1
		AC	SS	BCC	1
		AC	SS+IM	FCC+B2	1
		AC	SS	BCC	1
		AC	SS	BCC	1
		AC	SS	BCC	1
		AC	SS+IM	BCC+B2	1
		AC	SS	BCC	1
		AC	SS+IM	BCC+B2	1
		AC	SS+IM	BCC+B2	1
		AC	SS+IM	BCC+B2	19
	$AlCoCrFeNi_2$	AC	SS+IM	$BCC+B2+ L1_2$	20
	$AlCoCrFeNi_{2.1}$	AC	SS+IM	$BCC+B2+ L1_2$	20
		AC	SS+IM	FCC+B2	1
	$AlCoCrFeNi_{2.2}$	AC	SS+IM	$BCC+B2+ L1_2$	20
	$AlCoCr_2FeNi$	AC	SS+IM	$BCC+B2+ L1_2$	1
	$Al_{1.12}CoCrFeNi$	AC	SS+IM	BCC+B2	16
	$Al_{1.2}CoCrFeNi$	AC	SS+IM	BCC+B2	1
	$Al_{1.25}CoCrFeNi$	AC+1100C/24h/WQ	IM	B2	1
		AC	IM	B2	1
		AC+1100C/24h/WQ	SS+IM	BCC+B2	1
	$Al_{1.33}CoCrFeNi$	AC	SS+IM	BCC+B2	16
	$Al_{1.5}CoCrFeNi$	AC	IM	B2	1
		AC+1100C/24h/WQ	IM	BCC	1
		AC	IM	B2	1
		AC+1100C/24h/WQ	SS+IM	BCC+B2	1
		AC	IM	B2	1
		AC	SS+IM	BCC+B2	1
		AC	SS+IM	BCC+B2	19
	$Al_{1.71}CoCrFeNi$	AC	SS+IM	BCC+B2	15

		AC+1050C/120h	SS+IM	BCC+B2	15
	$Al_{1.8}CoCrFeNi$	AC	SS+IM	BCC+B2	1
	$Al_2CoCrFeNi$	AC	IM	B2	1
		AC	IM	B2	1
		AC+1100C/24h/WQ	SS+IM	BCC+B2	1
		AC+1100C/24h/WQ	IM	BCC	1
		AC	SS+IM	BCC+B2	1
		AC	IM	B2	1
		AC	SS+IM	BCC+B2	1
		AC	IM	B2	19
	$Al_{2.08}CoCrFeNi$	AC	SS+IM	BCC+B2	1
	$Al_{2.5}CoCrFeNi$	AC	IM	B2	1
		AC	SS+IM	BCC+B2	1
		AC	SS	BCC+B2	19
	$Al_3CoCrFeNi$	AC	IM	B2	1
		AC	IM	B2	1
		AC	IM	B2	19
	$Al_{10}Co_{21}Cr_{18}Fe_{36}Ni_{15}$	AC	IM+SS	FCC+B2	21
AlCoCrFeNiSi	$AlCoCrFeNiSi_{0.2}$	AC	SS	BCC	1
	$AlCoCrFeNiSi_{0.4}$	AC	SS	2BCC	1
	$AlCoCrFeNiSi_{0.6}$	AC	SS+IM	2BCC+IM	1
	$AlCoCrFeNiSi_{0.8}$	AC	SS+IM	2BCC+IM	1
	AlCoCrFeNiSi	AC	SS+IM	2BCC+IM	1
AlCoCrFeNiSiTi	$AlCoCrFe_6NiSiTi$	MA+LC	SS	BCC	22
	$AlCo_{0.6}CrFe_{0.2}Ni_{0.2}Si_{0.2}Ti_{0.2}$	AC	SS+IM	BCC+Cr3Si	23
	$AlCo_{0.2}CrFe_{0.2}NiSiTi_{0.2}$	AC	SS+IM	BCC+Cr3Si	23
	$AlCo_{0.2}Cr_{1.5}Fe_{0.2}NiSiTi_{0.2}$	AC	SS+IM	BCC+Cr3Si	23
AlCoCrFeNiTi	$Al_{0.2}Co_{1.5}CrFeNi_{1.5}Ti_{0.5}$	AC+1100C/4h/AC+800C/10h	SS	FCC	1
	$Al_{0.2}Co_{1.5}CrFeNi_{1.5}Ti$	AC+1100C/4h/AC+800C/10h	SS+IM	FCC+$D0_{24}$	1
	$Al_{0.3}CoCrFeNiTi_{0.1}$	AC	SS	FCC	1
		AC+700C/144h/WQ	SS+IM	FCC+B2	1
	$Al_{0.5}CoCrFeNiTi$	AC	SS+IM	BCC+B2+C14	1
		AC	SS+IM	BCC+FCC+B2+C14+$E9_3$	1
		AC+1000C/2h	SS+IM	BCC+FCC+B2+C14+$E9_3$+Unk	1
	$Al_{0.6}CoCrFeNiTi_{0.4}$	MA+SPS	SS+IM	2FCC+$D0_{22}$	1
	$Al_{0.6}CoCrFeNiTi$	AC	SS+IM	BCC+B2	24
	$Al_{0.75}CoCrFeNiTi_{0.25}$	MA+SPS	SS	BCC+FCC	25
	$AlCoCrFeNiTi_{0.5}$	AC	SS+IM	2BCC+B2	1

		AC	SS	2BCC	1
		AC	SS+IM	2BCC+B2	1
	AlCoCrFeNiTi	AC	SS+IM	2BCC+B2	1
		AC	SS+IM	BCC+B2+C14	1
		AC	SS+IM	BCC+FCC+B2+C14+ $E9_3$	1
		AC+1000C/2h	SS+IM	BCC+FCC+B2+C14+ $E9_3$+Unk	1
		MA+SPS	SS+IM	B2+FCC	26
	$AlCoCrFeNiTi_{1.5}$	AC	SS+IM	BCC+B2+C14	1
	$AlCo_{1.5}CrFeNiTi_{0.5}$	AC	SS	BCC+FCC	1
	$AlCo_2CrFeNiTi_{0.5}$	AC	SS	BCC+FCC	1
	$AlCo_3CrFeNiTi_{0.5}$	AC	SS	BCC+FCC	1
	$Al_{1.5}CoCrFeNiTi$	AC	SS+IM	BCC+B2	1
		AC	SS+IM	BCC+B2	1
		AC+1000C/2h	SS+IM	BCC+B2	1
	$Al_2CoCrFeNiTi$	AC	SS+IM	BCC+B2	1
		AC	SS+IM	BCC+B2	1
		AC+1000C/2h	SS+IM	BCC+B2	1
	$Al_4Co_{23.5}Cr_{23.5}Fe_{23.5}Ni_{23.5}Ti_2$	AC+1473K/4h	SS	FCC	27
		AC+1473K/4h+CR(30%)+1273K/2h+1073K/18h+WQ	SS+IM	FCC + $L1_2$	27
		AC+1473K/4h+CR(70%)+923K/4h+WQ	SS+IM	FCC + $L1_2$	27
	$Al_{12}Co_{20}Cr_{17}Fe_{35}Ni_{12}Ti_4$	AC	SS+IM	FCC+BCC+B2+ $L2_1$	21
AlCoCrNi	AlCoCrNi	AC	SS+IM	BCC+B2	28
AlCoCrNiSi	$Al_{0.43}CoCr_{0.29}NiSi_{0.14}$	AC	SS+IM	FCC+$L1_0$+$L1_2$	1
	$Al_{0.8}CoCrNiSi_{0.2}$	AC	SS+IM	BCC+B2+σ	1
AlCoCuFeGaNi	$Al_{0.4}CoCu_{0.4}FeGa_{0.02}Ni$	AC	SS	FCC+BCC	29
	$Al_{0.4}CoCu_{0.4}FeGa_{0.04}Ni$	AC	SS	FCC+BCC	29
	$Al_{0.4}CoCu_{0.4}FeGa_{0.06}Ni$	AC	SS	FCC+BCC	29
	$Al_{0.4}CoCu_{0.4}FeGa_{0.08}Ni$	AC	SS	FCC+BCC	29
AlCoCuFeNdNi	AlCoCuFeNdNi	AC	Unk	Unk	1
AlCoCuFeNi	$Al_{0.2}CoCu_{0.2}FeNi$	AC	SS	FCC	30
	$Al_{0.3}CoCu_{0.7}FeNi$	AC	SS	FCC	31
		MA+SPS	SS	FCC	31
	$Al_{0.4}CoCu_{0.4}FeNi$	AC	SS	FCC	30
		AC	SS	FCC+BCC	29
	$Al_{0.6}CoCu_{0.6}FeNi$	AC	SS	FCC	30
	$Al_{0.7}CoCu_{0.7}FeNi$	AC	SS	FCC+BCC	30
	$Al_{0.8}CoCu_{0.8}FeNi$	AC	SS+IM	BCC+ $L1_2$	30
	$Al_{0.9}CoCu_{0.9}FeNi$	AC	SS	FCC+BCC	30

	$Al_{1.2}CoCu_{1.2}FeNi$	AC	SS	FCC+BCC	30
	$AlCo_{0.2}CuFeNi$	AC	SS+IM	FCC+B2	1
	$AlCo_{0.5}CuFeNi$	AC	SS+IM	FCC+B2	1
	$AlCoCu_{0.33}FeNi$	AC+850C/24h	SS+IM	FCC+B2	32
	$AlCoCu_{0.5}FeNi$	AC	SS	BCC+FCC	1
	AlCoCuFeNi	AC	SS+IM	FCC+B2	1
		AC	SS	BCC+FCC	1
		AC+850C/24h	SS+IM	FCC+B2	32
		AC	SS	FCC+BCC	30
	$AlCo_{1.5}CuFeNi$	AC	SS+IM	FCC+B2	1
	$AlCo_{2}CuFeNi$	AC	SS+IM	FCC+B2	1
	$AlCo_{3}CuFeNi$	AC	SS	FCC	1
AlCoCuFeNiSi	AlCoCuFeNiSi	AC	SS	BCC+FCC	1
		AC	SS+IM	BCC+FCC+B2	9
AlCoCuFeNiTi	AlCoCuFeNiTi	AC	SS+IM	BCC+FCC+B2	1
		AC	SS	BCC+FCC	1
AlCoCuFeNiZr	AlCoCuFeNiZr	AC	SS+IM	$FCC+B2+D2_{b}$	1
AlCoCuNi	AlCoCuNi	AC	SS	BCC+FCC	1
AlCoCuNiTiZn	AlCoCuNiTiZn	MA+HP	SS	BCC+2FCC	1
		MA	SS	BCC + FCC	33
AlCoFeMnNi	AlCoFeMnNi	AC	IM	B2	34
		AC+1050C/50h+WQ	SS	BCC+FCC	34
	$Al_{0.25}CoFeMn_{0.25}Ni$	AC	SS	FCC	35
		AC+CR	SS	FCC	35
		AC+CR+900C/1h+WQ	SS+IM	FCC+B2	35
AlCoFeMoNi	$AlCoFeMo_{0.5}Ni$	AC	IM	B2+σ	1
AlCoFeNi	$Al_{0.25}CoFeNi$	AC	SS	FCC	1
	$Al_{0.5}CoFeNi$	AC	SS+IM	FCC+B2	1
	$Al_{0.75}CoFeNi$	AC	SS+IM	FCC+B2	1
	AlCoFeNi	AC	IM	B2	1
AlCoFeNiSi	$Al_{0.1}CoFeNiSi_{0.1}$	AC	SS	FCC	36
	$Al_{0.2}CoFeNiSi_{0.2}$	AC	SS	FCC	36
	$Al_{0.3}CoFeNiSi_{0.3}$	AC	SS	FCC+BCC	36
	$Al_{0.4}CoFeNiSi_{0.4}$	AC	SS	BCC	36
	$Al_{0.5}CoFeNiSi_{0.5}$	AC	SS	BCC	36
	$Al_{0.8}CoFeNiSi_{0.8}$	AC	SS	BCC	36
AlCoFeNiTi	$Al_{0.6}CoFeNiTi_{0.4}$	SPS	SS+IM	FCC+B2+Unk	1
	AlCoFeNiTi	AC	SS	BCC	1
AlCrCuFeMnNi	$Al_{0.3}CrCuFeMnNi$	AC	SS	BCC+2FCC	1
	$Al_{0.5}CrCuFeMnNi$	AC	SS	2BCC+FCC	1
	$Al_{0.8}CrCuFeMnNi$	AC	SS	2BCC+FCC	1
	$Al_{0.8}CrCuFeMn_{1.5}Ni$	AC	SS	2BCC	1

	$Al_{0.8}CrCuFe_{1.5}MnNi$	AC	SS	2BCC+FCC	1
	$Al_{0.8}CrCu_{1.5}FeMnNi$	AC	SS	BCC+FCC	1
	AlCrCuFeMnNi	AC	SS	2BCC	1
AlCrCuFeNi	$Al_{0.2}CrCuFeNi$	AC	SS	FCC	1
	$Al_{0.2}CrCuFeNi_2$	AC	SS	FCC	1
	$Al_{0.3}CrCuFeNi_2$	AC	SS	2FCC	37
		AC+CR(20%)+1000C/3h	SS	FCC	37
		AC+CR(20%)+1000C/3h+550C/150h	SS+IM	FCC+ $L1_2$	37
		AC+CR(20%)+1000C/3h+7000C/50h	SS+IM	FCC+ $L1_2$	37
		AC+CR(20%)+1000C/3h+8000C/20h	SS+IM	FCC+ $L1_2$	37
		AC+CR(20%)+1000C/3h+9000C/20h	SS+IM	FCC+ $L1_2$	37
	$Al_{0.4}CrCuFeNi_2$	AC	SS	FCC	1
		AC	SS	FCC	1
	$Al_{0.5}CrCuFeNi_2$	AC	SS	FCC	1
	$Al_{0.6}CrCuFeNi_2$	AC	SS	FCC	1
	$Al_{0.7}CrCuFeNi_2$	AC	SS	FCC	1
	$Al_{0.8}CrCuFeNi_2$	AC	SS	BCC+FCC	1
		AC	SS	FCC	1
	$Al_{0.9}CrCuFeNi_2$	AC	SS+IM	BCC+FCC+B2	1
	$AlCrCu_{0.5}FeNi$	AC	SS+IM	BCC+B2	1
	$AlCrCuFeNi_{0.6}$	AC	SS+IM	FCC+B2	1
	$AlCrCuFeNi_{0.8}$	AC	SS+IM	FCC+B2	1
	AlCrCuFeNi	AC	SS+IM	FCC+B2	1
	$AlCrCuFeNi_{1.2}$	AC	SS+IM	FCC+B2	1
	$AlCrCuFeNi_{1.4}$	AC	SS+IM	FCC+B2	1
	$AlCrCuFeNi_2$	AC	SS	BCC+FCC	1
		AC	SS+IM	BCC+FCC+B2	38
	$Al_{1.2}CrCuFeNi_2$	AC	SS+IM	BCC+FCC+B2	1
		AC	SS	BCC+FCC	1
	$Al_{1.5}CrCuFeNi_2$	AC	SS+IM	BCC+FCC+B2	1
	$Al_{1.8}CrCuFeNi_2$	AC	SS+IM	BCC+B2	1
	$Al_2CrCuFeNi_2$	AC	SS+IM	BCC+B2	1
	$Al_{2.2}CrCuFeNi_2$	AC	SS+IM	BCC+B2	1
	$Al_{2.5}CrCuFeNi_2$	AC	SS+IM	BCC+B2	1
AlCrCuFeNiTi	$AlCr_{0.5}CuFeNiTi$	AC	SS+IM	FCC+B2	1
	AlCrCuFeNiTi	AC	SS+IM	FCC+B2	1
		AC	SS+IM	2BCC+C14	1
		AC+500/4h/FC	SS+IM	2BCC+C14	1

	$AlCr_{1.5}CuFeNiTi$	AC	SS+IM	FCC+B2	1
	$AlCr_2CuFeNiTi$	AC	SS+IM	FCC+B2	1
	$AlCr_3CuFeNiTi$	AC	SS+IM	FCC+B2	1
AlCrFeMnNi	$Al_{0.1}CrFe_2Mn_{1.25}Ni_{0.75}$	AC	SS+IM	BCC+FCC+B2	39
	$Al_{0.3}CrFe_{1.5}MnNi_{0.5}$	AC	SS	BCC+FCC	1
		AC	SS+IM	BCC+FCC+B2	1
		AC+1100C/2h/FC	SS+IM	BCC+FCC+B2	1
		AC+700C/20h/FC	SS+IM	BCC+FCC+IM	1
		AC	SS	BCC+FCC	1
		AC+700C/20h	SS+IM	BCC+FCC+σ	1
	$Al_{0.32}CrFe_2Mn_{1.25}Ni_{0.75}$	AC	SS+IM	BCC+FCC+B2	39
	$Al_{0.5}CrFe_{1.5}MnNi_{0.5}$	AC	SS	BCC	1
		AC	SS+IM	2BCC+B2	1
		AC+FOR/1200C(50%)+1100C/6h/AC	SS+IM	2BCC+B2	1
		AC+700C/20h/FC	SS+IM	2BCC+B2+IM	1
		AC	SS	BCC	1
		AC+700C/20h	SS+IM	BCC+σ	1
	$Al_{0.56}CrFe_2Mn_{1.25}Ni_{0.75}$	AC	SS+IM	BCC+FCC+B2	39
	$Al_{0.8}CrFe_{1.5}MnNi_{0.5}$	AC	SS	BCC	1
		AC+700C/20h	SS	BCC	1
	$Al_{0.81}CrFe_2Mn_{1.25}Ni_{0.75}$	AC	SS+IM	BCC+FCC+B2	39
	$Al_{1.2}CrFe_{1.5}MnNi_{0.5}$	AC	SS	BCC	1
		AC+700C/20h	SS+IM	BCC+B2	1
	$Al_{10}Cr_{18}Fe_{36}Mn_{21}Ni_{15}$	AC	SS+IM	BCC+B2	40
		AC	SS+IM	FCC+BCC+B2	21
		AC+1200c/24h	SS+IM	BCC+B2	40
		AC+1000C/24h	SS+IM	BCC+FCC+B2	40
	$Al_{14.1}Cr_6Fe_{28.2}Mn_{32.9}Ni_{18.8}$	AC	SS+IM	FCC+B2	41
AlCrFeMnNiTi	AlCrFeMnNiTi	MA	SS	BCC+FCC	42
	$Al_{12}Cr_{17}Fe_{35}Mn_{20}Ni_{12}Ti_4$	AC	IM+SS	BCC+ $L2_1$	21
AlCrFeMnTi	$AlCrFeMnTi_{0.25}$	AC	SS+IM	BCC+ $L2_1$	43
	$Al_{1.5}CrFeMnTi$	AC	SS+IM	BCC+ $L2_1$+C14	43
	$Al_2CrFeMnTi_{0.25}$	AC	SS+IM	BCC+ $L2_1$	43
	$Al_2CrFeMnTi$	AC	SS+IM	BCC+ $L2_1$+ C14	43
	$Al_3CrFeMnTi_{0.25}$	AC	SS+IM	BCC+$L2_1$+IM	43
	$Al_4CrFeMnTi_{0.25}$	AC	SS+IM	BCC+ $L2_1$+IM	43
AlCrFeMoNi	$AlCrFeMo_{0.2}Ni$	AC	SS+IM	BCC+B2	1
	$AlCrFeMo_{0.5}Ni$	AC	SS+IM	BCC+B2	1
	$AlCrFeMo_{0.8}Ni$	AC	IM	B2+σ	1
	AlCrFeMoNi	AC	IM	B2+σ	1
AlCrFeNi	$Al_{0.15}CrFe_{1.5}Ni_{0.5}$	AC	SS	BCC	1

	$Al_{0.2}CrFe_{1.5}Ni_{0.5}$	AC	SS	BCC	1
	$Al_{0.3}CrFe_{1.5}Ni_{0.5}$	AC	IM	B2	1
	$Al_{0.4}CrFe_{1.5}Ni_{0.5}$	AC	IM	B2	1
	$Al_{0.6}CrFeNi_{2.4}$	AC	SS	FCC	44
	$Al_{0.7}CrFeNi_{2.3}$	AC	SS+IM	BCC+FCC+B2	44
	$Al_{0.75}CrFeNi$	MA	SS	BCC+FCC	25
	$Al_{0.8}CrFeNi_{2.2}$	AC	SS+IM	BCC+FCC+B2	44
	$Al_{0.9}CrFeNi$	AC	SS+IM	BCC+B2	45
	$Al_{0.9}CrFeNi_{2.1}$	AC	SS+IM	BCC+FCC+B2	44
	AlCrFeNi	AC	SS+IM	BCC+B2	1
		AC	SS+IM	BCC+B2	45
	$AlCrFeNi_{0.75}$	AC+850C/24h	SS+IM	BCC+B2	32
	$AlCrFeNi_{2}$	AC	SS+IM	BCC+FCC+B2	44
	$AlCrFe_{2}Ni_{2}$	AC	SS+IM	BCC+FCC+B2	46
	$Al_{1.1}CrFeNi$	AC	SS+IM	BCC+B2	45
	$Al_{1.2}CrFeNi$	AC	SS+IM	BCC+B2	45
	$Al_{1.3}CrFeNi$	AC	SS+IM	BCC+B2	45
AlCrFeNiTiV	$Al_{0.5}CrFeNiTiV$	AC+700C/20h	IM	C15+$L2_{1}$	1
AlCrMnNbV	AlCrMnNbV	Not Mentioned	IM	Laves+Unk	47
AlCrMnNbTi	AlCrMnNbTi	Not Mentioned	IM	Laves+Unk	47
AlCrMoNbSiTiV	$Al_{0.5}CrMoNbSi_{0.3}TiV$	AC	SS+IM	BCC+IM	48
AlCrMoNbTi	$Al_{0.5}CrMoNbTi$	AC	SS	BCC	48
	AlCrMoNbTi	AC	SS	BCC	49
		AC+1300C/20h	SS	BCC	50
		AC+1100C/20h	SS+IM	BCC+C14	49
		AC+1200C/20h	SS+IM	BCC+C14	49
		AC+1300C/20h	SS+IM	BCC+C14	49
AlCrMoNbTiV	$Al_{0.5}CrMoNbTiV$	AC	SS	BCC	48
AlCrMoNbV	$Al_{0.5}CrMoNbV$	AC	SS	BCC	48
AlCrMoSiTi	AlCrMoSiTi	AC	IM	B2+$D8_{m}$	1
AlCrMoTi	AlCrMoTi	AC+1200C/20h	SS	BCC	50
AlCrNbTiV	$Al_{0.5}CrNbTi_{2}V_{0.5}$	AC	SS	BCC	51
		AC+1200C/24h	SS+IM	BCC+C14	51
	$AlCr_{0.5}NbTiV$	AC+1200C/24h	SS	BCC	52
		AC+1200C/24h+HP/800C	SS+IM	BCC+C14	52
		AC+1200C/24h+HP/1000C	SS+IM	BCC+C14	52
	AlCrNbTiV	AC+1200C/24h	SS+IM	BCC+C14	52
		AC+1200C/24h+HP/800C	SS+IM	BCC+C14	52
		AC+1200C/24h+HP/1000C	SS+IM	BCC+C14	52
	$AlCr_{1.5}NbTiV$	AC+1200C/24h	SS+IM	BCC+C14	52
		AC+1200C/24h+HP/800C	SS+IM	BCC+C14	52
		AC+1200C/24h+HP/1000C	SS+IM	BCC+C14	52

AlCuLiMgSn	$AlCu_{0.5}Li_{0.5}MgSn_{0.2}$	AC	SS+IM	A5+$D0_2$+2IM	1
AlCuLiMgZn	$AlCu_{0.2}Li_{0.5}MgZn_{0.5}$	AC	IM	IM	1
	$Al_{16}CuLiMgZn$	AC	SS+IM	FCC+C16+IM	1
AlCuNi	AlCuNi	AC	SS+IM	FCC+B2	1
AlCuNiPdTiZr	$Al_{0.5}CuNiPdTiZr$	MS	SS+IM	BCC+B2	53
AlHfNbTaTiZr	$Al_{0.3}HfNbTaTiZr$	AC	SS	BCC	54
	$Al_{0.4}Hf_{0.6}NbTaTiZr$	AC+HP/1200C/207MPa/2h +1200C/24h/FC	SS	BCC	1
		AC+HP/1200C/207MPa/2h +1200C/24h/FC	SS	BCC	1
	$Al_{0.5}HfNbTaTiZr$	AC	SS	BCC	54
	$Al_{0.75}HfNbTaTiZr$	AC	SS	BCC	54
	AlHfNbTaTiZr	AC	SS	2BCC	54
AlLiMgSnZn	$AlLi_{0.5}MgSn_{0.2}Zn_{0.5}$	AC	SS+IM	FCC+$D0_2$+2IM	1
	AlLiMgSnZn	AC	SS+IM	A5+FCC+HCP+$D0_2$+IM	1
	$Al_{16}LiMgSnZn$	AC	SS+IM	A5+FCC+$D0_2$+IM	1
AlMnNbTiV	AlMnNbTiV	Not Mentioned	IM	B2+Laves	47
AlMoNbTaTiZr	$AlMo_{0.5}NbTa_{0.5}TiZr$	AC+HP/1400C/207MPa/2h +1400C/24h/FC	SS	2BCC	1
		AC+HP/1400C/207MPa/2h +1400C/24h/FC	SS	2BCC	55
		AC+HP/1400C/207MPa/2h +1400C/24h/FC	SS+IM	BCC+B2	1
		AC+HP/1400C/207MPa/2h +1400C/24h/FC	SS+IM	BCC+B2	1
AlMoNbTi	AlMoNbTi	AC+1500C/20h	SS	BCC	50
AlMoNbTiV	$Al_{0.25}MoNbTiV$	AC	SS	BCC	56
	$Al_{0.5}MoNbTiV$	AC	SS	BCC	56
	$Al_{0.75}MoNbTiV$	AC	SS	BCC	56
	AlMoNbTiV	AC	SS	BCC	56
	$Al_{1.5}MoNbTiV$	AC	SS+IM	BCC+B2	56
AlMoTaTiV	$Al_{0.2}MoTaTiV$	AC	SS	BCC	57
	$Al_{0.6}MoTaTiV$	AC	SS	BCC	57
	AlMoTaTiV	AC	SS	BCC	57
AlNbTaTiV	$Al_{0.25}NbTaTiV$	AC	SS	BCC	1
	$Al_{0.5}NbTaTiV$	AC	SS	BCC	1
	AlNbTaTiV	AC	SS	BCC	1
AlNbTaTiVZr	$Al_{0.3}NbTa_{0.8}Ti_{1.4}V_{0.2}Zr_{1.3}$	AC+HP/1200C/207MPa/2h +1200C/24h/FC	SS	BCC	1
	$Al_{0.5}NbTa_{0.8}Ti_{1.5}V_{0.2}Zr$	AC+HP/1200C/207MPa/2h +1200C/24h/FC	SS	2BCC	1

		AC+HP/1200C/210MPa/2h +1200C/24h/FC	SS+IM	BCC+B2	58
		AC+HP/1200C/210MPa/2h +1200C/24h/FC+1200C/2h/ WQ	SS+IM	BCC+B2	58
		AC+HP/1200C/210MPa/2h +1200C/24h/FC+1200C/2h/ WQ+800C/1000C/20h/WQ	SS+IM	BCC+IM	58
		AC+HP/1200C/210MPa/2h +1200C/24h/FC+1200C/2h/ WQ+600C/120h/WQ	SS+IM	BCC+B2+IM	58
		AC+HP/1200C/210MPa/2h +1200C/24h/FC+600C/120 h/WQ	SS+IM	BCC+B2	58
AlNbTaTiZr	$Al_{0.3}NbTaTi_{1.4}Zr_{1.3}$	AC+HP/1200C/207MPa/2h +1200C/24h/FC	SS	2BCC	1
	$AlNb_{1.5}Ta_{0.5}Ti_{1.5}Zr_{0.5}$	AC+HP/1400C/207MPa/2h +1400C/24h/FC	SS	BCC	1
AlNbTiV	AlNbTiV	AC+1200C/24h	SS	BCC	52
		AC+1200C/24h+HP/800C	SS+IM	BCC+C14	52
		AC+1200C/24h+HP/1000C	SS+IM	BCC+C14	52
		AC	SS	BCC	59
AlNbTiVZr	$Al_{0.24}NbTiVZr$	AC+1400C/276MPa/2h+14 00C/6h	SS+IM	BCC+C14	60
	$Al_{0.5}NbTiVZr$	AC	SS+IM	BCC+C14+Zr2Al	61
	$Al_{1.5}NbTiVZr$	AC	SS+IM	BCC+ C14+Zr2Al	61
	AlNbTiVZr	AC	SS+IM	BCC+ C14+Zr2Al	61
	$AlNbTiVZr_{0.5}$	AC	SS+IM	BCC+C14+Zr2Al	62
AlTiVYZr	AlTiVYZr	AC	IM	Unk	1
BeCuNiTiVZr	BeCuNiTiVZr	AC	IM	Unk	1
CoCrCuFeMn	CoCrCuFeMn	AC+850C/72h	SS	2FCC	1
CoCrCuFeMnNi	CoCrCuFeMnNi	AC	SS	FCC	1
	CoCrCuFeMnNi	AC	SS	2FCC	2
	$CoCrCu_2FeMnNi$	AC	SS	2FCC	2
CoCrCuFeMnNiTiV	CoCrCuFeMnNiTiV	AC	SS+IM	2BCC+FCC+σ+Unk	1
CoCrCuFeNi	$CoCrCu_{0.2}FeNi$	AC	SS	FCC	63
	$CoCrCu_{0.4}FeNi$	AC	SS	FCC	63
	$CoCrCu_{0.5}FeNi$	AC	SS	FCC	1
		AC	SS	2FCC	1
		AC	SS	3FCC	1
		AC+1050C/1h+1250C/24h	SS	3FCC	1
		AC+1050C/1h+1350C/24h	SS	2FCC	1
		AC	SS	3FCC	1

		AC+1050C/1h+1250/24h/WQ	SS	3FCC	1
		AC+1050C/1h+1350/24h/WQ	SS	3FCC	1
	$CoCrCu_{0.6}FeNi$	AC	SS	FCC	63
	$CoCrCu_{0.8}FeNi$	AC	SS	FCC	63
	CoCrCuFeNi	AC	SS	FCC	1
		AC	SS	FCC	1
		AC	SS	FCC	1
		AC	SS	FCC	1
		AC	SS	2FCC	1
		AC	SS	FCC	1
		MA+SPS	SS+IM	2FCC+σ	1
		AC	SS	FCC+BCC	19
CoCrCuFeNiTi	$CoCrCuFeNiTi_{0.5}$	AC	SS	2FCC	1
		AC	SS	2FCC	1
	$CoCrCuFeNiTi_{0.8}$	AC	SS+IM	FCC+C14	1
	CoCrCuFeNiTi	AC	SS+IM	FCC+C14	1
	$CoCrCuFeNiTi_{2.5}$	AC	IM	Unk	1
CoCrCuNi	CoCrCuNi	MA	SS	FCC	64
CoCrFeGaNi	CoCrFeGaNi	AC	SS	FCC+BCC	65
CoCrFeGeMnNi	CoCrFeGeMnNi	AC	SS	BCC+FCC	1
CoCrFeGeNi	CoCrFeGeNi	AC	SS	FCC+BCC	65
CoCrFeMn	$Co_{0.25}Cr_{0.25}FeMn$	AC	SS	FCC	1
		AC+HR/900C(50%)+1200C/2h/WQ	SS	FCC	1
	$CoCrFe_{3.5}Mn_{4.5}$	AC+HR/900C(50%)+1200C/2h/WQ	SS	FCC	66
	$CoCrFe_{4}Mn_{4}$	AC+HR/900C(50%)+1200C/2h/WQ	SS	FCC	66
	$CoCrFe_{4.5}Mn_{3.5}$	AC+HR/900C(50%)+1200C/2h/WQ	SS	FCC	66
	CoCrFe5Mn3	AC+HR/900C(50%)+1200C/2h/WQ+CR(60%)+900C/3min	SS	FCC+HCP	66
CoCrFeMnNbNi	CoCrFeMnNbNi	AC	SS	FCC	1
CoCrFeMnNi	$Co_{0.5}CrFeMn_{1.5}Ni$	AC+700C/20h	SS+IM	FCC+σ	1
	$CoCr_{0.4}Fe_{8}Mn_{5.4}Ni_{5.2}$	AC	SS	FCC	1
		AC+HR/900C(50%)+1200C/2h/WQ	SS	FCC	1
		AC+HR/900C(50%)+1200C/2h/WQ+CR(64%)+900C/10min	SS	FCC	1

	$CoCr_{0.75}FeMn_{0.75}Ni$	AC+700C/20h	SS	FCC	1
	$CoCrFe_{0.5}Mn_{0.5}Ni_{1.5}$	AC+700C/20h	SS	FCC	1
	CoCrFeMnNi	AC	SS	FCC	1
		AC+1000C/72h	SS	FCC	1
		AC	SS	FCC	1
		AC+1200/24h/WQ	SS	FCC	1
		AC+1200C/48h	SS	FCC	1
		AC+1200C/48h+HPT+450 C/5min	SS+IM	BCC+FCC+IM	1
		AC+1200C/48h+HPT+450 C/1h	SS+IM	BCC+FCC+IM	1
		AC+1200C/48h+HPT+450 C/15h	SS+IM	BCC+FCC+2IM	1
		AC+1200C/48h+HPT+600 C/1h	SS+IM	FCC+IM	1
		AC+1200C/48h+HPT+700 C/1h	SS+IM	FCC+IM	1
		AC+1200C/48h+HPT+800 C/1h	SS	FCC	1
		AC+1000C/24h+CR(80%)+ 1100C/1h	SS	FCC	1
		AC+1000C/24h+CR(80%)+ 575C/1h	SS	FCC	1
		AC	SS	FCC	1
		AC+1000C/24h	SS	FCC	1
		AC+900C/2h	SS	FCC	67
		AC	SS	FCC	68
		AC+1000C/24h	SS	FCC	68
	$CoCrFe_{2.7}MnNi$	AC+900C/2h	SS	FCC	67
	$CoCrFe_{6}MnNi$	AC+900C/2h	SS	FCC	67
	$CoCr_{1.25}FeMn_{0.25}Ni$	AC+700C/20h	SS	FCC	1
	$CoCr_{1.3}FeMnNi_{0.7}$	AC+1000C/24h+CR(80%)+ 1100C/1h	SS	FCC	1
		AC+1000C/24h+CR(80%)+ 800C/1h	SS	FCC	1
		AC+1000C/24h+CR(80%)+ 700C/1h	SS+IM	FCC+σ	1
		AC+1000C/24h+CR(80%)+ 575C/1h	SS+IM	FCC+σ	1
	$Co_{1.4}CrFeMnNi$	AC+1000C/24h+CR(80%)+ 1100C/1h	SS	FCC	1
		AC+1000C/24h+CR(80%)+ 575C/1h	SS	FCC	1

	$Co_{1.5}Cr_{0.5}FeMn_{0.5}Ni$	AC+700C/20h	SS	FCC	1
	$Co_5Cr_2Fe_{40}Mn_{27}Ni_{26}$	AC	SS	FCC	69
		AC+1200C/2h	SS	FCC	69
CoCrFeMnNiTi	CoCrFeMnNiTi	AC	SS	BCC+FCC	1
CoCrFeMnNiV	$CoCrFeMnNiV_{0.25}$	AC	SS	FCC	1
		AC+1000C/24h	SS+IM	FCC+σ	1
	$CoCrFeMnNiV_{0.5}$	AC	SS+IM	FCC+σ	1
		AC+1000C/24h	SS+IM	FCC+σ	1
	$CoCrFeMnNiV_{0.75}$	AC	SS+IM	FCC+σ	1
		AC+1000C/24h	SS+IM	FCC+σ	1
	CoCrFeMnNiV	AC	SS	BCC+FCC	1
		AC	SS+IM	FCC+σ	1
		AC+1000C/24h	SS+IM	FCC+σ	1
		AC	SS+IM	FCC+σ	68
		AC+1000C/24h	SS+IM	FCC+σ	68
	$CoCr_{1.5}Fe_{3.5}Mn_{0.5}Ni_{2.5}V$	AC+1100C/6h	SS	FCC	70
		AC+1100C/6h+CR(75%)+900C/10min+WQ	SS	FCC	70
		AC+1100C/6h+CR(75%)+750C/10min+WQ	SS	FCC	70
CoCrFeMoNi	$CoCrFeMo_{0.3}Ni$	AC	SS	FCC	1
		AM	SS+IM	FCC+σ	1
	$CoCrFeMo_{0.5}Ni$	AM	SS+IM	FCC+σ	1
	$CoCrFeMo_{0.85}Ni$	AM	SS+IM	FCC+σ+$D8_5$	1
	$Co_{42.5}Cr_{12.5}Fe_{20}Mo_5Ni_{20}$	AC+1200C/48h	SS	FCC	71
	$Co_{35}Cr_{15}Fe_{20}Mo_{10}Ni_{20}$	AC+1200C/48h	SS+IM	FCC+$D8_5$	71
		AC+1200C/48h+HR/1100C	SS+IM	FCC+$D8_5$	71
		AC+1200C/48h+HR/1100C+800c/1h	SS+IM	FCC+$D8_5$	71
		AC+1200C/48h+HR/1100C+850c/5min/WQ	SS+IM	FCC+$D8_5$	71
		AC+1200C/48h+HR/1100C+900c/5min/WQ	SS+IM	FCC+$D8_5$	71
		AC+1200C/48h+HR/1100C+1000c/1h	SS+IM	FCC+$D8_5$	71
		AC+1200C/48h+HR/1100C+1150c/1h	SS	FCC	71
	$Co_{27.5}Cr_{17.5}Fe_{20}Mo_{15}Ni_{20}$	AC+1200C/48h	SS+IM	FCC+σ	71
	CoCrFeMoNi	AC+1200C/48h	SS+IM	FCC+σ	71
CoCrFeMoNiTi	$Co_{1.5}CrFeMo_{0.1}Ni_{1.5}Ti_{0.5}$	AC	SS	FCC	1
	$Co_{1.5}CrFeMo_{0.5}Ni_{1.5}Ti_{0.5}$	AC	SS+IM	FCC+σ	1
	$Co_{1.5}CrFeMo_{0.8}Ni_{1.5}Ti_{0.5}$	AC	SS+IM	FCC+σ	1

CoCrFeMoNiW	$CoCrFeMo_{0.5}NiW_{0.5}$	MA	SS	BCC+FCC	72
CoCrFeNbNi	$CoCrFeNb_{0.103}Ni$	AC	SS+IM	FCC+C14	1
	$CoCrFeNb_{0.155}Ni$	AC	SS+IM	FCC+C14	1
	$CoCrFeNb_{0.206}Ni$	AC	SS+IM	FCC+C14	1
	$CoCrFeNb_{0.309}Ni$	AC	SS+IM	FCC+C14	1
	$CoCrFeNb_{0.412}Ni$	AC	SS+IM	FCC+C14	1
CoCrFeNbNi	$CoCrFeNb_{0.25}Ni$	AC	SS+IM	FCC+Laves	73
		AC+600C/7days	SS+IM	FCC+Laves	73
		AC+750C/7days	SS+IM	FCC+Laves	73
		AC+900C/7days	SS+IM	FCC+Laves	73
		AC+1000C/7days	SS+IM	FCC+Laves	73
CoCrFeNi	CoCr0.5FeNi	AC	SS	FCC	74
	CoCr0.6FeNi	AC	SS	FCC	74
	CoCr0.7FeNi	AC	SS	FCC	74
	CoCr0.8FeNi	AC	SS	FCC	74
	CoCr0.95FeNi	AC	SS	FCC	74
	CoCr0.9FeNi	AC	SS	FCC	74
	CoCrFeNi	AC	SS	2FCC	1
		AC	SS	FCC	1
		AC+1100C/24h/WQ	SS	FCC	1
		AC+1100C/24h/WQ+CR(50%)	SS	FCC	1
		AC+1100C/24h/WQ	SS	FCC	1
		AC+CR	SS	FCC	1
		AC	SS	FCC	1
		AC	SS	FCC	1
		MA+SPS	SS+IM	FCC+σ	1
		AC+1200/24h/WQ	SS	FCC	1
		AM	SS	FCC	1
		AM+750C/12h/WQ	SS	FCC	1
		AM+1000C/12h/WQ	SS	FCC	1
		AC	SS	FCC	1
		AC+1000C/24h+CR(80%)+1100C/1h	SS	FCC	1
		AC+1000C/24h+CR(80%)+575C/1h	SS	FCC	1
		AC	SS	FCC	68
		AC+1000C/24h	SS	FCC	68
		AC	SS	FCC	75
	$CoCr_{1.05}FeNi$	AC	SS	FCC	74
	$CoCr_{1.10}FeNi$	AC	SS	FCC	74
	$CoCr_{1.15}FeNi$	AC	SS	FCC	74

	$CoCr_2FeNi$	AC+700C/20h	SS+IM	FCC+σ	1
CoCrFeNiPd	CoCrFeNiPd	AC+CR	SS	FCC	1
	$CoCrFeNiPd_2$	AC+CR	SS	FCC	1
CoCrFeNiSn	CoCrFeNiSn	Induction Melting	SS	FCC+BCC	65
CoCrFeNiTi	$Co_{0.5}CrFeNiTi_{0.5}$	MA+SPS	SS+IM	FCC+σ	1
	$CoCrFeNiTi_{0.3}$	AC	SS+IM	$FCC+HCP+L1_2$	1
		AC	SS+IM	FCC+C14+IM+σ	1
		AC+600C/6h/FC	SS+IM	FCC+C14+IM+σ	1
		AC+700C/6h/FC	SS+IM	FCC+C14+IM+σ	1
		AC+800C/6h/FC	SS+IM	FCC+C14+IM+σ	1
		AC+1000C/6h/FC	SS+IM	FCC+IM+σ	1
		AC	SS	FCC	75
	$CoCrFeNiTi_{0.5}$	AC	SS+IM	FCC+C36+σ+R	75
	CoCrFeNiTi	AC	SS	FCC	1
		MA	SS	FCC	76
		AC	SS+IM	$BCC+FCC+E9_3$	1
		AC+1000C/2h	SS+IM	$BCC+FCC+C14+E9_3+$ Unk	1
	$Co_{1.5}CrFeNi_{1.5}Ti_{0.5}$	AC	SS	FCC	1
		AC+1100C/4h/AC+800C/10h/AC	SS+IM	$FCC+D0_{24}$	1
	$Co_{1.5}CrFeNi_{1.5}Ti$	AC+1100C/4h/AC+800C/10h/AC	SS+IM	$FCC+D0_{24}$	1
CoCrFeNiV	CoCrFeNiV	AC	SS+IM	FCC+σ	68
		AC+1000C/24h	SS+IM	FCC+σ	68
CoCrFeNiW	$CoCrFeNiW_{0.3}$	AC	SS	FCC	77
	CoCrFeNiW	MA	SS	BCC+FCC	72
CoCrMnNi	CoCrMnNi	AC+1100/24h/WQ	SS	FCC	1
		AC+900C/2h	SS	FCC	67
CoCrMnNiV	CoCrMnNiV	AC+1000C/72h	SS+IM	FCC+σ	1
CoCrMoNb	CoCrMoNb	AC	SS+IM	BCC+Laves	78
CoCrMoNbTi	$CoCrMoNbTi_{0.2}$	AC	SS+IM	BCC+Laves	78
	$CoCrMoNbTi_{0.4}$	AC	SS	BCC	78
	$CoCrMoNbTi_{0.5}$	AC	SS+IM	BCC+Laves	78
	CoCrMoNbTi	AC	SS+IM	BCC+Laves	78
CoCrMoNiW	$Co_2CrMo_{0.5}Ni_2W_{0.5}$	AC	SS+IM	$FCC+D8_5+σ$	79
CoCrNi	CoCrNi	AC+1200/24h/WQ	SS	FCC	1
CoCrNiVW	$Co_2Cr_{0.5}Ni_2VW_{0.5}$	AC	SS+IM	FCC+Laves	79
CoCuFeMnNi	CoCuFeMnNi	AC	SS	FCC	1
CoCuFeMnNiSn	$CoCuFeMnNiSn_{0.03}$	AC	SS	FCC	1
	$CoCuFeMnNiSn_{0.05}$	AC	SS+IM	FCC+IM	1
	$CoCuFeMnNiSn_{0.08}$	AC	SS+IM	FCC+IM	1

	$CoCuFeMnNiSn_{0.1}$	AC	SS+IM	FCC+IM	1
	$CoCuFeMnNiSn_{0.2}$	AC	SS+IM	FCC+IM	1
CoCuFeNi	CoCuFeNi	MA+SPS	SS	2FCC	1
		AC	SS	FCC	1
CoCuFeNiSn	$CoCuFeNiSn_{0.04}$	AC	SS+IM	FCC+IM	1
	$CoCuFeNiSn_{0.05}$	AC	SS+IM	FCC+IM	1
	$CoCuFeNiSn_{0.07}$	AC	SS+IM	FCC+IM	1
	$CoCuFeNiSn_{0.1}$	AC	SS+IM	FCC+IM	1
	$CoCuFeNiSn_{0.2}$	AC	SS+IM	FCC+IM	1
	$CoCuFeNiSn_{0.5}$	AC	SS+IM	FCC+IM	1
CoCuFeNiTi	CoCuFeNiTi	AC	SS	FCC	1
CoCuFeNiV	CoCuFeNiV	AC	SS	FCC	1
CoCuHfTiZr	CoCuHfTiZr	MS	AM	AM	1
CoFeMgNiTiZr	$Co_{0.5}Fe_{0.5}MgNi_{0.5}TiZr$	MA	SS	BCC	80
CoFeMnMoNi	CoFeMnMoNi	AC+1000C/72h	SS+IM	$FCC+D8_5$	1
CoFeMnNi	CoFeMnNi	AC+1100/24h/WQ	SS	FCC	1
CoFeMnNiV	CoFeMnNiV	AC+1000C/72h	SS+IM	FCC+σ	1
CoFeMnTiVZr	$CoFeMnTi_{0.5}VZr$	AC	IM	C14	1
	$CoFeMnTiV_{0.4}Zr$	AC	IM	C14	1
	$CoFeMnTiV_{0.7}Zr$	AC	IM	C14	1
	$CoFeMnTiVZr_{0.4}$	AC	IM	C14	1
	$CoFeMnTiVZr_{0.7}$	AC	IM	C14	1
	CoFeMnTiVZr	AC	IM	C14	1
	$CoFeMnTiVZr_{1.3}$	AC	IM	C14	1
	$CoFeMnTiVZr_{1.6}$	AC	IM	C14	1
	$CoFeMnTiVZr_2$	AC	IM	C14	1
	$CoFeMnTiVZr_{2.3}$	AC	IM	C14	1
	$CoFeMnTiVZr_{2.6}$	AC	IM	C14	1
	$CoFeMnTiVZr_3$	AC	IM	C14	1
	$CoFeMnTiV_{1.3}Zr$	AC	IM	C14	1
	$CoFeMnTiV_{1.6}Zr$	AC	IM	C14	1
	$CoFeMnTiV_2Zr$	AC	IM	C14	1
	$CoFeMnTiV_{2.3}Zr$	AC	IM	C14	1
	$CoFeMnTiV_{2.6}Zr$	AC	IM	C14	1
	$CoFeMnTiV_3Zr$	AC	IM	C14	1
	$CoFeMnTi_{1.5}VZr$	AC	IM	C14	1
	$CoFeMnTi_2VZr$	AC	IM	C14	1
	$CoFeMnTi_{2.5}VZr$	AC	IM	C14	1
CoFeNi	CoFeNi	MA+SPS	SS	FCC	1
		AC	SS	FCC	1
		AC	SS	FCC	1
		AC	SS	FCC	30

		AC+1200/24h/WQ	SS	FCC	1
		AC+850C/24h	SS	FCC	32
CoFeNiSi	$CoFeNiSi_{0.25}$	AC	SS	FCC	1
	$CoFeNiSi_{0.5}$	AC	SS+IM	$FCC+Ni_3Si$	1
	$CoFeNiSi_{0.75}$	AC	SS+IM	$FCC+Ni_3Si$	1
CoFeNiTi	CoFeNiTi	AC	Unk	FCC+Unk	1
CoFeNbNiV	$CoFeNb_{0.75}Ni_2V_{0.5}$	AC	SS+IM	FCC+ Laves	81
		AC+500C/6h	SS+IM	FCC+ Laves	81
		AC+600C/6h	SS+IM	FCC+NbNi4+Laves	81
		AC+700C/6h	SS+IM	FCC+NbNi4+Laves	81
		AC+800C/6h	SS+IM	FCC+NbNi4+Laves	81
		AC+1000C/6h	SS+IM	FCC+NbNi4+Laves	81
CoFeReRu	CoFeReRu	AC	SS	HCP	82
CoMnNi	CoMnNi	AC+1100/24h/WQ	SS	FCC	1
CoMoNiVW	$Co_2Mo_{0.5}Ni_2VW_{0.5}$	AC	SS+IM	FCC+BCC+Laves	79
CrCuFeMnNi	$Cr_{0.5}CuFeMn_{0.5}Ni$	AC	SS	2FCC	1
	$Cr_{0.5}CuFeMnNi_{0.5}$	AC	SS	BCC+2FCC	1
	$CrCu_{0.5}FeMnNi$	AC	SS	2FCC	1
	$CrCuFe_{0.5}Mn_{0.5}Ni$	AC	SS	BCC+2FCC	1
	$CrCuFe_{0.5}MnNi_{0.5}$	AC	SS	BCC+FCC	1
	CrCuFeMnNi	AC	SS	BCC+2FCC	1
		AC	SS	BCC+2FCC	1
	$CrCuFeMn_2Ni_2$	AC	SS	FCC	1
	Cr_2CuFe_2MnNi	AC	SS	BCC+2FCC	1
CrCuFeNi	$CrCu_{0.7}FeNi$	AC	SS	2FCC	83
CrCuFeNiZr	CrCuFeNiZr	AC	SS+IM	BCC+Unk	19
CrFeMnNi	$Cr_{0.66}FeMnNi$	AC+1200C/24h/WQ	SS	FCC	1
		AC+1200C/24h/WQ+CR(86%)+900C/1h	SS	FCC	1
	$CrFe_{1.5}MnNi_{0.5}$	AC	SS+IM	BCC+σ	1
		AC	SS+IM	FCC+σ	1
		AC+700C/20h	SS+IM	FCC+σ	1
	$CrFe_2Mn_{1.25}Ni_{0.75}$	AC	SS	BCC+FCC	39
CrFeMnNiTi	CrFeMnNiTi	AC+1000C/72h	SS+IM	A12+BCC+C14+Unk	1
		MA+700C	SS+IM	2FCC+σ	76
CrFeNi	CrFeNi	AC+1200/24h/WQ	SS	FCC	1
CrFeNiTi	CrFeNiTi	MA	SS+IM	2FCC+σ	76
CrFeNiV	$Cr_{0.75}FeNi_{2.25}V$	AC+1100C/6h+1000C/10min	SS	FCC	84
		AC+1100C/6h+950C/10min	SS+IM	FCC+σ	84

		AC+1100C/6h+900C/10min	SS+IM	FCC+σ	84
		AC+1100C/6h+800C/10min	SS+IM	FCC+σ	84
CrFeNiVW	$CrFeNiV_{0.5}W_{0.25}$	AC	SS+IM	FCC+σ	85
	$CrFeNiV_{0.5}W_{0.5}$	AC	SS+IM	FCC+BCC+σ	85
	$CrFeNiV_{0.5}W_{0.75}$	AC	SS+IM	FCC+BCC+σ	85
	$CrFeNiV_{0.5}W$	AC	SS+IM	FCC+BCC+σ	85
	$CrFeNi_2V_{0.5}W_{0.25}$	AC	SS+IM	FCC+σ	85
	$CrFeNi_2V_{0.5}W_{0.5}$	AC	SS+IM	FCC+BCC+σ	85
	$CrFeNi_2V_{0.5}W_{0.75}$	AC	SS+IM	FCC+BCC+σ	85
	$CrFeNi_2V_{0.5}W$	AC	SS+IM	FCC+BCC+σ	85
CrHfNbTiZr	CrHfNbTiZr	AC	SS+IM	BCC+Laves	86
		AC+1173K/600s	SS+IM	BCC+Laves	86
		AC+973K/600s	SS+IM	BCC+Laves	86
		AC+773K/600s	SS+IM	BCC+Laves	86
		AC+573K/600s	SS+IM	BCC+Laves	86
CrMnNbTiV	CrMnNbTiV	Not Mentioned	IM	Laves + Unk	47
CrMoNbTaTiVWZr	CrMoNbTaTiV1W1Zr	AC	SS	BCC	87
CrMoNbTaTiZr	$CrMo_{0.5}NbTa_{0.5}TiZr$	AC	SS+IM	2BCC+C15	1
		AC+HP/1450C/207MPa/3h	SS+IM	2BCC+C15	1
	CrMoNbTaTiVZr	AC	SS	BCC	87
CrMoNbTaVW	$Cr_{0.5}MoNbTaVW$	AC	SS	BCC	88
	CrMoNbTaVW	AC	SS	BCC	88
	$Cr_2MoNbTaVW$	AC	SS	2BCC	88
CrNbTiVZr	CrNbTiVZr	AC+HP/1200C/207MPa/2h +1200C/24h/FC	SS+IM	BCC+C15	1
CrNbTiZr	CrNbTiZr	AC+HP/1200C/207MPa/2h +1200C/24h/FC	SS+IM	BCC+C15	1
CrTaVW	CrTaVW	SPS	SS+IM	BCC+Laves	89
CrTaTiVW	$Cr_{0.23}Ta_{0.24}Ti_{0.04}V_{0.24}W_{0.25}$	SPS	SS+IM	BCC+Laves	89
	$Cr_{0.22}Ta_{0.23}Ti_{0.07}V_{0.23}W_{0.24}$	SPS	SS+IM	BCC+Laves	89
CuFeHfTiZr	CuFeHfTiZr	MS	AM	AM	1
CuHfNiTiZr	CuHfNiTiZr	MS	AM	AM	1
		AC	AM	AM	1
DyGdHoTbY	DyGdHoTbY	AC	SS	HCP	90
DyGdHoLuTb	DyGdHoLuTb	AC	SS	HCP	91
DyGdLuTbTm	DyGdLuTbTm	AC	SS	HCP	1
DyGdLuTbY	DyGdLuTbY	AC	SS	HCP	1
FeMnNi	FeMnNi	AC+1100/24h/WQ	SS	FCC	1
HfMoNbSiTiV	$HfMoNbSi_{0.3}TiV$	AC	SS+IM	BCC+Silicide	92
	$HfMoNbSi_{0.5}TiV$	AC	SS+IM	BCC+Silicide	92

	$HfMoNbSi_{0.7}TiV$	AC	SS+IM	BCC+Silicide	92
HfMoNbTaTi	HfMoNbTaTi	AC	SS	BCC	93
HfMoNbTaTiVZr	HfMoNbTaTiVZr	AC	SS	2BCC	94
HfMoNbTaTiVWZr	HfMoNbTaTiVWZr	AC	SS+IM	2BCC+C15+Unk	94
HfMoNbTaTiZr	$HfMo_{0.25}NbTaTiZr$	AC	SS	BCC	95
	$HfMo_{0.5}NbTaTiZr$	AC	SS	BCC	95
	$HfMo_{0.75}NbTaTiZr$	AC	SS	BCC	95
	HfMoNbTaTiZr	AC	SS	BCC	95
		AC	SS	BCC	93
		AC	SS	BCC	96
HfMoNbTaZr	HfMoNbTaZr	AC	SS	BCC	93
HfMoNbTiV	$HfMo_{0.5}NbTiV_{0.5}$	AC	SS	BCC	92
HfMoNbTiZr	$Hf_{0.5}Mo_{0.5}NbTiZr$	AC	SS	BCC	97
	$HfMo_{0.5}Nb_{0.5}TiZr$	AC	SS	BCC	98
	HfMoNbTiZr	AC	SS	BCC	99
		AC	SS	BCC	93
		AC+1373K/10h	SS	BCC	99
HfMoTaTiZr	HfMoTaTiZr	AC	SS	BCC	96
		AC	SS	BCC	93
HfMoTiVZr	$HfMo_{0.2}Ti_2V_{0.5}Zr$	AC	SS	BCC	100
HfNbTaTiVZr	HfNbTaTiV1Zr	AC	SS	BCC	101
HfNbTaTiZr	$Hf_{0.5}Nb_{0.5}Ta_{0.5}Ti_{1.5}Zr$	AC	SS	BCC	102
	$Hf_{0.75}NbTa_{0.5}Ti_{1.5}Zr_{1.25}$	AC	SS	BCC	103
	$Hf_{0.8}Nb_{0.31}Ta_{0.31}Ti_{0.18}Zr_{0.13}$	AC	SS	BCC	104
	$HfNb_{0.18}Ta_{0.18}Ti_{1.27}Zr$	AC+CR+900C/30min+600 C/30min	SS	BCC	105
	$HfNb_{0.5}Ta_{0.5}TiZr$	AC	SS	BCC	98
		AC+HP/800C/3min	SS	BCC	98
	HfNbTaTiZr	AC+1200C/24h	SS	BCC	106
		AC+1200C/24H+600C/100 h	SS	BCC+HCP	106
		AC+1200C/24H+800C/100 h	SS	BCC+HCP	106
		AC	SS	BCC	93
		AC+CR(70%)+1200C/10mi n	SS	BCC	107
		AC	SS	BCC	95
		AC	SS	BCC	54
		AC+CR(70%)+1200C/10mi n+1000C/96h	SS	BCC	107
		AC+CR(70%)+1200C/10mi n+900C/96h	SS	BCC	107

		AC+CR(70%)+1200C/10min+700C/2.5h	SS	2BCC+HCP	107
		AC+CR(70%)+1200C/10min+700C/96h	SS	2BCC+HCP	107
		AC+CR(70%)+1200C/10min+700C/152h	SS	2BCC+HCP	107
		AC+CR(70%)+1200C/10min+550C/96h	SS	2BCC+HCP	107
		AC+HP/1200C/207MPa/3h	SS	BCC+Unk	1
		AC+HP/1200C/207MPa/2h+1200C/24h+CR(86%)+800C/2h/FC	SS	2BCC	1
		AC+HP/1200C/207MPa/2h+1200C/24h+CR(86%)+1000C/2h/FC	SS	BCC	1
		AC+HP/1200C/207MPa/2h+1200C/24h+CR(86%)+1200C/2h/FC	SS	BCC	1
	$HfNb_3Ta_3TiZr$	AC	SS	BCC	108
HfNbTaZr	HfNbTaZr	AC	SS	BCC	109
		AC+1800C/48h	SS	BCC	109
		AC+1800C/4 days	SS	BCC+HCP	109
		AC+1800C/8 days	SS	BCC+HCP	109
HfNbTiVZr	$HfNb_2TiVZr_2$	AC	SS	BCC	98
	$HfNb_{0.5}TiV_{0.5}Zr$	AC	SS	BCC	98
	HfNbTiVZr	AC	SS	BCC	110
		AC	SS	BCC	111
		AC	SS	BCC	86
		AC+1173K/600s	SS	BCC+Laves	86
		AC+973K/600s	SS	BCC	86
		AC+773K/600s	SS	BCC	86
		AC+573K/600s	SS	BCC	86
HfNbTiZr	HfNbTiZr	AC	SS	BCC	1
		AC+1300C/6h/FC	SS	BCC	1
HfNbZr	HfNbZr	AC	SS	BCC	112
		SP	SS	BCC	112
HfTaTiZr	$HfTa_{0.4}TiZr$	AC	SS	BCC+HCP	113
	$HfTa_{0.5}TiZr$	AC	SS	BCC+HCP	113
	$HfTa_{0.6}TiZr$	AC	SS	BCC+HCP	113
	HfTaTiZr	AC	SS	BCC	113
LaPbSeSnTe	$La_{0.02}Pb_{0.98}SeSnTe$	AC+873K/120h+MA+SPS	SS	FCC	114
	$La_{0.04}Pb_{0.96}SeSnTe$	AC+873K/120h+MA+SPS	SS	FCC	114
	$La_{0.06}Pb_{0.94}SeSnTe$	AC+873K/120h+MA+SPS	SS	FCC	114

	$La_{0.08}Pb_{0.92}SeSnTe$	AC+873K/120h+MA+SPS	SS	FCC	114
	$La_{0.1}Pb_{0.9}SeSnTe$	AC+873K/120h+MA+SPS	SS	FCC	114
MoNbTaTiV	MoNbTaTiV	AC	SS	BCC	115
MoNbTaTiVW	MoNbTaTiVW	AC	SS	BCC	116
		AC	SS	BCC	117
		AC+1200C/24h	SS	BCC	117
MoNbTaTiVZr	MoNbTaTiVZr	AC	SS	BCC	87
MoNbTaTiW	$MoNbTaTi_{0.25}W$	AC	SS	BCC	118
	$MoNbTaTi_{0.5}W$	AC	SS	BCC	118
	$MoNbTaTi_{0.75}W$	AC	SS	BCC	118
	MoNbTaTiW	AC	SS	BCC	118
		AC	SS	BCC	117
		AC+1200C/24h	SS	BCC	117
MoNbTaTiZr	MoNbTaTiZr	AC	SS	2BCC	119
		AC	SS	2BCC	120
		AC+1273K/168h	SS	2BCC	120
	MoNbTaV	AC	SS	BCC	121
MoNbTaVW	MoNbTaVW	AC	SS	BCC	1
		AC	SS	BCC	1
		AC+1400C/19h	SS	BCC	1
		AC	SS	BCC	122
MoNbTaW	MoNbTaW	AC	SS	BCC	122
		AC	SS	BCC	1
		AC	SS	BCC	1
		AC	SS	BCC	118
		AC+1400C/19h	SS	BCC	1
MoNbTiV	MoNbTiV	AC	SS	BCC	56
MoNbTiVZr	$Mo_{0.1}NbTiV_{0.3}Zr$	AC	SS	BCC	123
	$Mo_{0.3}NbTiV_{0.3}Zr$	AC	SS	BCC	123
		AC+1273K/72h	SS	BCC	123
	$Mo_{0.3}NbTiVZr$	AC	SS	BCC	123
	$Mo_{0.5}NbTiV_{0.3}Zr$	AC	SS	BCC	123
	$Mo_{0.5}NbTiVZr$	AC	SS	BCC	123
	$Mo_{0.7}NbTiV_{0.3}Zr$	AC	SS	BCC	123
		AC+1273K/72h	SS+IM	2BCC+C15	123
	$Mo_{0.7}NbTiVZr$	AC	SS	BCC	123
	$MoNbTiV_{0.25}Zr$	AC	SS	BCC	1
	$MoNbTiV_{0.3}Zr$	AC	SS	BCC	123
	$MoNbTiV_{0.5}Zr$	AC	SS	BCC	1
	$MoNbTiV_{0.75}Zr$	AC	SS	BCC	1
	MoNbTiVZr	AC	SS	BCC	1
		AC	SS	BCC	123

		AC+1400C/276MPa/2h+1400C/6h	SS	2BCC+C15	60
	$MoNbTiV_{1.5}Zr$	AC	SS	2BCC	1
	$MoNbTiV_2Zr$	AC	SS	2BCC	1
	$MoNbTiV_3Zr$	AC	SS	2BCC	1
	$Mo_{1.3}NbTiV_{0.3}Zr$	AC	SS	BCC	123
	$Mo_{1.3}NbTiVZr$	AC	SS	2BCC	123
	$Mo_{1.5}NbTiV_{0.3}Zr$	AC	SS	BCC	123
	$Mo_{1.5}NbTiVZr$	AC	SS	2BCC	123
	$Mo_{1.7}NbTiVZr$	AC	SS	2BCC	123
	$Mo_2NbTiVZr$	AC	SS	2BCC	123
MoNbTiZr	MoNbTiZr	AC	SS	2BCC	1
MoPdRhRu	MoPdRhRu	AC+1700C/20h	SS	HCP	1
MoRhRu	MoRhRu	AC+1700C/20h	SS	HCP	1
MoTaTiV	MoTaTiV	AC	SS	BCC	57
MoTaTiVZr	MoTaTiVZr	AC	SS	BCC	87
NbTaTiV	NbTaTiV	AC	SS	BCC	1
		AC	SS	BCC	124
	NbTaTiVZr	AC+HP/1400C/276MPa/2h+1400C/6h	SS	BCC	60
NbTaTiVW	NbTaTiVW	AC	SS	BCC	124
NbTaTiZr	NbTaTiZr	AC+HP/1773K/150MPa/2h+1773K/2h+WQ	SS	BCC	125
		AC	SS	BCC	120
NbTaVW	NbTaVW	AC	SS	BCC	124
NbTiVZr	$NbTiV_{0.3}Zr$	AC	SS	BCC	123
	NbTiVZr	AC+HP/1200C/207MPa/2h+1200C/24h/FC	SS	BCC	1
		AC+1400C/276MPa/2h+1400C/6h	SS	BCC	60
		AC	SS	BCC	123
		AC	SS+IM	BCC+HCP+Unk	87
	$NbTiV_2Zr$	AC+HP/1200C/207MPa/2h+1200C/24h/FC	SS	3BCC	1
NbTiZr	NbTiZr	AC+1400C/276MPa/2h+1400C/6h	SS	BCC	60
PbSeSnTe	PbSeSnTe	AC+873K/120h+MA+SPS	SS	FCC	114

Section 2: XRD patterns for the newly synthesized HEAs

Fig.S1 includes representative XRD patterns for some of the newly synthesized HEAs.

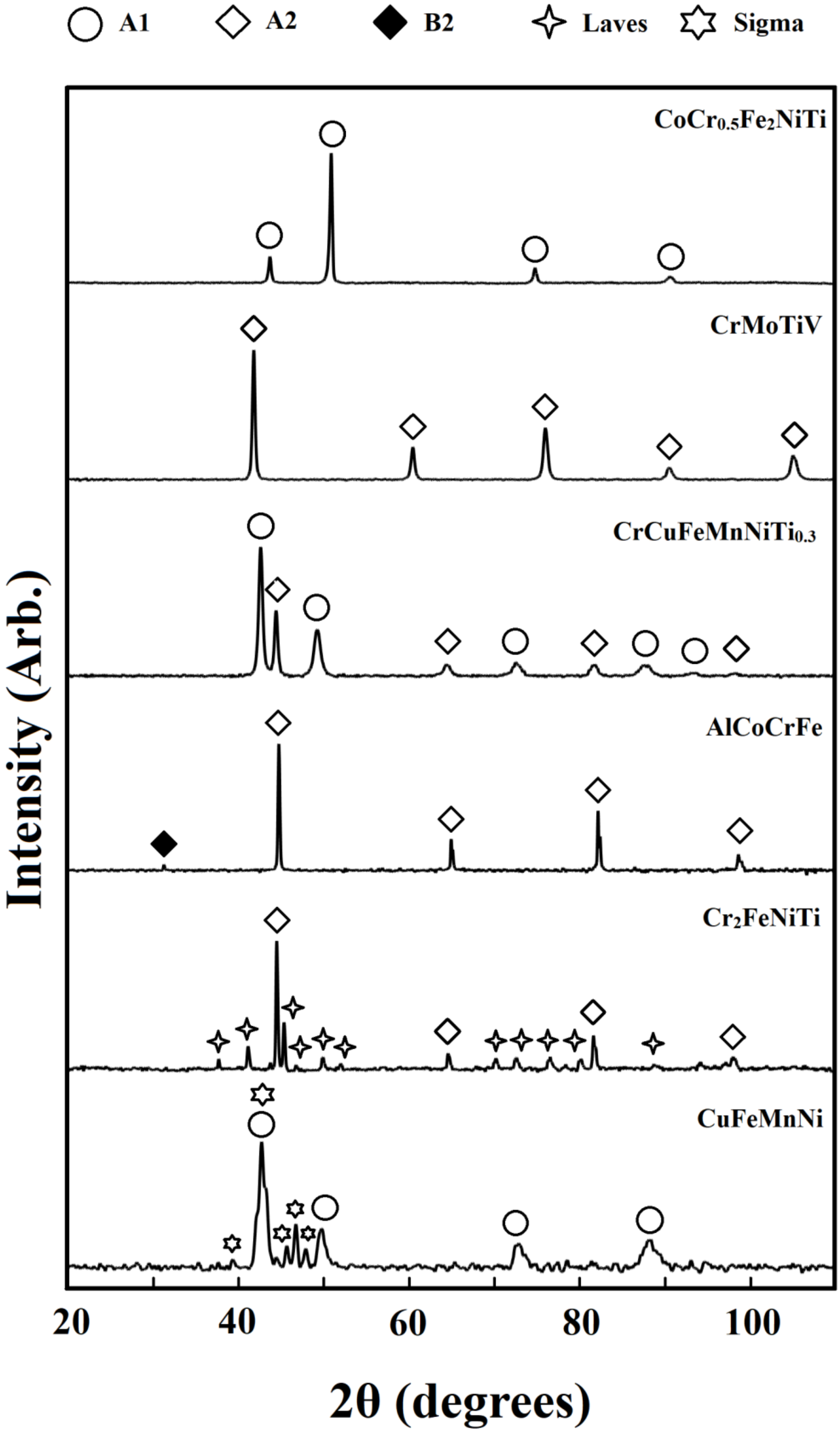

Fig. S1: XRD patterns for some newly synthesized HEAs.

Reference:

1. Miracle, D. B. & Senkov, O. N. A critical review of high entropy alloys and related concepts. *Acta Mater.* **122**, 448–511 (2017).
2. Nagase, T. Microstructure of Co-Cr-Fe-Mn-Ni-Cu and Co-Cr-Fe-Mn-Ni-Ag High Entropy Alloys with Liquid Phase Separation. *Mater. Sci. Forum* **941**, 1238–1241 (2018).
3. Wang, Z. G. *et al.* Effect of coherent L1 2 nanoprecipitates on the tensile behavior of a fcc-based high-entropy alloy. *Mater. Sci. Eng. A* **696**, 503–510 (2017).
4. Wu, C.-S., Tsai, P.-H., Kuo, C.-M. & Tsai, C.-W. Effect of Atomic Size Difference on the Microstructure and Mechanical Properties of High-Entropy Alloys. *Entropy* **20**, 967 (2018).
5. KE, G.-Y., Chen, S., HSU, T. & Yeh, J. FCC and BCC equivalents in as-cast solid solutions of AlxCo yCrzCu0.5FevNiw high-entropy alloys. *Ann. Chim. Sci. des Mater.* **31**, 669–683 (2006).
6. Manzoni, A. *et al.* Investigation of phases in Al23Co15Cr 23Cu8Fe15Ni16 and Al 8Co17Cr17Cu8Fe17Ni 33 high entropy alloys and comparison with equilibrium phases predicted by Thermo-Calc. *J. Alloys Compd.* **552**, 430–436 (2013).
7. Yang, C. C., Hang Chau, J. L., Weng, C. J., Chen, C. S. & Chou, Y. H. Preparation of high-entropy AlCoCrCuFeNiSi alloy powders by gas atomization process. *Mater. Chem. Phys.* **202**, 151–158 (2017).
8. Zhang, S., Wu, C. L. & Zhang, C. H. Phase evolution characteristics of FeCoCrAlCuVxNi high entropy alloy coatings by laser high-entropy alloying. *Mater. Lett.* **141**, 7–9 (2015).
9. Wang, X. R., Wang, Z. Q., Lin, T. S., He, P. & Sekulic, D. P. Impact of CrSiTi and NiSi on the Thermodynamics, Microstructure, and Properties of AlCoCuFe-Based High-Entropy Alloys. *J. Mater. Eng. Perform.* **25**, 2053–2064 (2016).
10. Hsu, C. Y., Wang, W. R., Tang, W. Y., Chen, S. K. & Yeh, J. W. Microstructure and mechanical properties of new AlCoxCrFeMo 0.5Ni high-entropy alloys. *Adv. Eng. Mater.* **12**, 44–49 (2010).
11. Juan, C. C. *et al.* On microstructure and mechanical performance of AlCoCrFeMo 0.5Ni x high-entropy alloys. *Intermetallics* **32**, 401–407 (2013).
12. Joseph, J. *et al.* Comparative study of the microstructures and mechanical properties of direct laser fabricated and arc-melted AlxCoCrFeNi high entropy alloys. *Mater. Sci. Eng. A* **633**, 184–193 (2015).
13. Yasuda, H. Y., Miyamoto, H., Cho, K. & Nagase, T. Formation of ultrafine-grained microstructure in Al0.3CoCrFeNi high entropy alloys with grain boundary precipitates. *Mater. Lett.* **199**, 120–123 (2017).

14. Gwalani, B. *et al.* Optimizing the coupled effects of Hall-Petch and precipitation strengthening in a Al0.3CoCrFeNi high entropy alloy. *Mater. Des.* **121**, 254–260 (2017).
15. Butler, T. & Weaver, M. Influence of Annealing on the Microstructures and Oxidation Behaviors of Al8(CoCrFeNi)92, Al15(CoCrFeNi)85, and Al30(CoCrFeNi)70 High-Entropy Alloys. *Metals (Basel).* **6**, 222 (2016).
16. Ma, Y. *et al.* The BCC/B2 Morphologies in AlxNiCoFeCr High-Entropy Alloys. *Metals (Basel).* **7**, 57 (2017).
17. Wang, Q. *et al.* A cuboidal B2 nanoprecipitation-enhanced body-centered-cubic alloy Al0.7CoCrFe2Ni with prominent tensile properties. *Scr. Mater.* **120**, 85–89 (2016).
18. Hao, J. *et al.* Formation of cuboidal B2 nanoprecipitates and microstructural evolution in the body-centered-cubic Al 0.7 NiCoFe 1.5 Cr 1.5 high-entropy alloy. *J. Alloys Compd.* **780**, 408–421 (2019).
19. Li, C., Li, J. C., Zhao, M. & Jiang, Q. Effect of alloying elements on microstructure and properties of multiprincipal elements high-entropy alloys. *J. Alloys Compd.* **475**, 752–757 (2009).
20. Lu, Y. *et al.* Directly cast bulk eutectic and near-eutectic high entropy alloys with balanced strength and ductility in a wide temperature range. *Acta Mater.* **124**, 143–150 (2017).
21. Stepanov, N. D., Shaysultanov, D. G., Tikhonovsky, M. A. & Zherebtsov, S. V. Structure and high temperature mechanical properties of novel non-equiatomic Fe-(Co, Mn)-Cr-Ni-Al-(Ti) high entropy alloys. *Intermetallics* **102**, 140–151 (2018).
22. Zhang, H., Pan, Y., He, Y. & Jiao, H. Microstructure and properties of 6FeNiCoSiCrAlTi high-entropy alloy coating prepared by laser cladding. *Appl. Surf. Sci.* **257**, 2259–2263 (2011).
23. Wang, L. M., Chen, C. C., Yeh, J. W. & Ke, S. T. The microstructure and strengthening mechanism of thermal spray coating NixCo0.6Fe0.2CrySi zAlTi0.2 high-entropy alloys. *Mater. Chem. Phys.* **126**, 880–885 (2011).
24. Chen, L. *et al.* Wear behavior of HVOF-sprayed Al 0.6 TiCrFeCoNi high entropy alloy coatings at different temperatures. *Surf. Coatings Technol.* **358**, 215–222 (2019).
25. Chen, Z. *et al.* Effects of Co and Ti on microstructure and mechanical behavior of Al0.75FeNiCrCo high entropy alloy prepared by mechanical alloying and spark plasma sintering. *Mater. Sci. Eng. A* **648**, 217–224 (2015).
26. Zhang, K. B. *et al.* Characterization of nanocrystalline CoCrFeNiTiAl high-entropy solid solution processed by mechanical alloying. *J. Alloys Compd.* **495**, 33–38 (2010).
27. He, J. Y. *et al.* A precipitation-hardened high-entropy alloy with outstanding tensile properties. *Acta Mater.* **102**, 187–196 (2016).
28. Jumaev, E. *et al.* Chemical evolution-induced strengthening on AlCoCrNi dual-phase high-entropy alloy with high specific strength. *J. Alloys Compd.* **777**, 828–834 (2019).

29. Li, Z. *et al.* Correlation between the magnetic properties and phase constitution of FeCoNi(CuAl)0.8Gax (0 ≤ x ≤ 0.08) high-entropy alloys. *J. Alloys Compd.* **746**, 285–291 (2018).
30. Zhang, Q. *et al.* The effects of phase constitution on magnetic and mechanical properties of FeCoNi(CuAl) x (x = 0–1.2) high-entropy alloys. *J. Alloys Compd.* **693**, 1061–1067 (2017).
31. Fu, Z. *et al.* Microstructure and strengthening mechanisms in an FCC structured single-phase nanocrystalline Co25Ni25Fe25Al7.5Cu17.5high-entropy alloy. *Acta Mater.* **107**, 59–71 (2016).
32. Singh, A. K. & Subramaniam, A. On the formation of disordered solid solutions in multi-component alloys. *J. Alloys Compd.* **587**, 113–119 (2014).
33. Varalakshmi, S., Kamaraj, M. & Murty, B. S. Processing and properties of nanocrystalline CuNiCoZnAlTi high entropy alloys by mechanical alloying. *Mater. Sci. Eng. A* **527**, 1027–1030 (2010).
34. Karati, A., Guruvidyathri, K., Hariharan, V. S. & Murty, B. S. Thermal stability of AlCoFeMnNi high-entropy alloy. *Scr. Mater.* **162**, 465–467 (2019).
35. Li, P., Wang, A. & Liu, C. T. A ductile high entropy alloy with attractive magnetic properties. *J. Alloys Compd.* **694**, 55–60 (2017).
36. Zhang, Y., Zuo, T., Cheng, Y. & Liaw, P. K. High-entropy alloys with high saturation magnetization, electrical resistivity, and malleability. *Sci. Rep.* **3**, 1–7 (2013).
37. Gwalani, B. *et al.* Cu assisted stabilization and nucleation of L12 precipitates in Al0.3CuFeCrNi2 fcc-based high entropy alloy. *Acta Mater.* **129**, 170–182 (2017).
38. Ma, S. G. *et al.* Strain rate effects on the dynamic mechanical properties of the AlCrCuFeNi2 high-entropy alloy. *Mater. Sci. Eng. A* **649**, 35–38 (2016).
39. Stepanov, N. D., Shaysultanov, D. G., Chernichenko, R. S., Tikhonovsky, M. A. & Zherebtsov, S. V. Effect of Al on structure and mechanical properties of Fe-Mn-Cr-Ni-Al non-equiatomic high entropy alloys with high Fe content. *J. Alloys Compd.* **770**, 194–203 (2019).
40. Shaysultanov, D. G. *et al.* Novel Fe36Mn21Cr18Ni15Al10high entropy alloy with bcc/B2 dual-phase structure. *J. Alloys Compd.* **705**, 756–763 (2017).
41. Baker, I., Meng, F., Wu, M. & Brandenberg, A. Recrystallization of a novel two-phase FeNiMnAlCr high entropy alloy. *J. Alloys Compd.* **656**, 458–464 (2016).
42. Mishra, R. K., Sahay, P. P. & Shahi, R. R. Alloying, magnetic and corrosion behavior of AlCrFeMnNiTi high entropy alloy. *J. Mater. Sci.* **54**, 4433–4443 (2019).
43. Feng, R. *et al.* Design of light-weight high-entropy alloys. *Entropy* **18**, 16–29 (2016).
44. Jin, X. *et al.* A new CrFeNi2Al eutectic high entropy alloy system with excellent mechanical properties. *J. Alloys Compd.* **770**, 655–661 (2019).
45. Chen, X. *et al.* Effects of aluminum on microstructure and compressive properties of Al-Cr-Fe-Ni eutectic multi-component alloys. *Mater. Sci. Eng. A*

681, 25–31 (2017).
46. Dong, Y., Gao, X., Lu, Y., Wang, T. & Li, T. A multi-component AlCrFe2Ni2 alloy with excellent mechanical properties. *Mater. Lett.* **169**, 62–64 (2016).
47. Ye, Y. F., Wang, Q., Lu, J., Liu, C. T. & Yang, Y. High-entropy alloy: challenges and prospects. *Mater. Today* **19**, 349–362 (2016).
48. Liu, C. M., Wang, H. M., Zhang, S. Q., Tang, H. B. & Zhang, A. L. Microstructure and oxidation behavior of new refractory high entropy alloys. *J. Alloys Compd.* **583**, 162–169 (2014).
49. Chen, H. *et al.* Microstructure and mechanical properties at elevated temperatures of a new Al-containing refractory high-entropy alloy Nb-Mo-Cr-Ti-Al. *J. Alloys Compd.* **661**, 206–215 (2016).
50. Chen, H. *et al.* Contribution of Lattice Distortion to Solid Solution Strengthening in a Series of Refractory High Entropy Alloys. *Metall. Mater. Trans. A Phys. Metall. Mater. Sci.* **49**, 772–781 (2018).
51. Stepanov, N. D., Yurchenko, N. Y., Panina, E. S., Tikhonovsky, M. A. & Zherebtsov, S. V. Precipitation-strengthened refractory Al 0.5 CrNbTi 2 V 0.5 high entropy alloy. *Mater. Lett.* **188**, 162–164 (2016).
52. Stepanov, N. D., Yurchenko, N. Y., Skibin, D. V., Tikhonovsky, M. A. & Salishchev, G. A. Structure and mechanical properties of the AlCr<inf>x</inf>NbTiV (x = 0, 0.5, 1, 1.5) high entropy alloys. *J. Alloys Compd.* **652**, 266–280 (2015).
53. Nagase, T., Takeuchi, A., Amiya, K. & Egami, T. Solid state amorphization of metastable Al0.5TiZrPdCuNi high entropy alloy investigated by high voltage electron microscopy. *Mater. Chem. Phys.* **210**, 291–300 (2018).
54. Lin, C. M., Juan, C. C., Chang, C. H., Tsai, C. W. & Yeh, J. W. Effect of Al addition on mechanical properties and microstructure of refractory AlxHfNbTaTiZr alloys. *J. Alloys Compd.* **624**, 100–107 (2015).
55. Senkov, O. N., Senkova, S. V. & Woodward, C. Effect of aluminum on the microstructure and properties of two refractory high-entropy alloys. *Acta Mater.* **68**, 214–228 (2014).
56. Chen, S. Y., Yang, X., Dahmen, K. A., Liaw, P. K. & Zhang, Y. Microstructures and crackling noise of AlxNbTiMoV high entropy alloys. *Entropy* **16**, 870–884 (2014).
57. Qiao, D. X., Jiang, H., Chang, X. X., Lu, Y. P. & Li, T. J. Microstructure and Mechanical Properties of $VTaTiMoAl_x$ Refractory High Entropy Alloys. *Mater. Sci. Forum* **898**, 638–642 (2017).
58. Soni, V. *et al.* Phase stability as a function of temperature in a refractory high-entropy alloy. *J. Mater. Res.* **33**, 3235–3246 (2018).
59. Stepanov, N. D., Shaysultanov, D. G., Salishchev, G. A. & Tikhonovsky, M. A. Structure and mechanical properties of a light-weight AlNbTiV high entropy alloy. *Mater. Lett.* **142**, 153–155 (2015).
60. Senkov, O. N., Rao, S., Chaput, K. J. & Woodward, C. Compositional effect on microstructure and properties of NbTiZr-based complex concentrated alloys. *Acta Mater.* **151**, 201–215 (2018).

61. Stepanov, N. D., Yurchenko, N. Y., Shaysultanov, D. G., Salishchev, G. A. & Tikhonovsky, M. A. Effect of Al on structure and mechanical properties of Al x NbTiVZr (x = 0, 0.5, 1, 1.5) high entropy alloys . *Mater. Sci. Technol.* **31**, 1184–1193 (2015).
62. Stepanov, N. D., Yurchenko, N. Y., Sokolovsky, V. S., Tikhonovsky, M. A. & Salishchev, G. A. An AlNbTiVZr0.5 high-entropy alloy combining high specific strength and good ductility. *Mater. Lett.* **161**, 136–139 (2015).
63. Verma, A. *et al.* High temperature wear in CoCrFeNiCux high entropy alloys: The role of Cu. *Scr. Mater.* **161**, 28–31 (2019).
64. Durga, A., Hari Kumar, K. C. & Murty, B. S. Phase formation in equiatomic high entropy alloys: CALPHAD approach and experimental studies. *Trans. Indian Inst. Met.* **65**, 375–380 (2012).
65. Vida, A. *et al.* Effects of the sp element additions on the microstructure and mechanical properties of NiCoFeCr based high entropy alloys. *Mater. Sci. Eng. A* **669**, 14–19 (2016).
66. Li, Z., Pradeep, K. G., Deng, Y., Raabe, D. & Tasan, C. C. Metastable high-entropy dual-phase alloys overcome the strength-ductility trade-off. *Nature* **534**, 227–30 (2016).
67. Ondicho, I. *et al.* Experimental investigation and phase diagram of CoCrMnNi–Fe system bridging high-entropy alloys and high-alloyed steels. *J. Alloys Compd.* **785**, 320–327 (2019).
68. Salishchev, G. A. *et al.* Effect of Mn and V on structure and mechanical properties of high-entropy alloys based on CoCrFeNi system. *J. Alloys Compd.* **591**, 11–21 (2014).
69. Yao, M. J., Pradeep, K. G., Tasan, C. C. & Raabe, D. A novel, single phase, non-equiatomic FeMnNiCoCr high-entropy alloy with exceptional phase stability and tensile ductility. *Scr. Mater.* **72–73**, 5–8 (2014).
70. Jo, Y. H. *et al.* Cryogenic strength improvement by utilizing room-temperature deformation twinning in a partially recrystallized VCrMnFeCoNi high-entropy alloy. *Nat. Commun.* **8**, 1–8 (2017).
71. Ming, K., Bi, X. & Wang, J. Precipitation strengthening of ductile Cr 15 Fe 20 Co 35 Ni 20 Mo 10 alloys. *Scr. Mater.* **137**, 88–93 (2017).
72. Shang, C. *et al.* CoCrFeNi(W 1 − x Mo x) high-entropy alloy coatings with excellent mechanical properties and corrosion resistance prepared by mechanical alloying and hot pressing sintering. *Mater. Des.* **117**, 193–202 (2017).
73. He, F. *et al.* Strengthening the CoCrFeNiNb 0.25 high entropy alloy by FCC precipitate. *J. Alloys Compd.* **667**, 53–57 (2016).
74. Lucas, M. S. *et al.* Thermomagnetic analysis of FeCoCrxNi alloys: Magnetic entropy of high-entropy alloys. *J. Appl. Phys.* **113**, 1–4 (2013).
75. Shun, T. T., Chang, L. Y. & Shiu, M. H. Microstructures and mechanical properties of multiprincipal component CoCrFeNiTi x alloys. *Mater. Sci. Eng. A* **556**, 170–174 (2012).
76. Mishra, R. K. & Shahi, R. R. Phase evolution and magnetic characteristics of

TiFeNiCr and TiFeNiCrM (M = Mn, Co) high entropy alloys. *J. Magn. Magn. Mater.* **442**, 218–223 (2017).
77. Poletti, M. G., Fiore, G., Gili, F., Mangherini, D. & Battezzati, L. Development of a new high entropy alloy for wear resistance: FeCoCrNiW0.3and FeCoCrNiW0.3+ 5 at.% of C. *Mater. Des.* **115**, 247–254 (2017).
78. Zhang, M., Zhou, X. & Li, J. Microstructure and Mechanical Properties of a Refractory CoCrMoNbTi High-Entropy Alloy. *J. Mater. Eng. Perform.* **26**, 3657–3665 (2017).
79. Jiang, H. *et al.* Microstructure and Mechanical Properties of the W-Ni-Co System Refractory High-Entropy Alloys. *Mater. Sci. Forum* **816**, 324–329 (2015).
80. Co, M. *et al.* ScienceDirect Hydrogen-induced phase transition of. *Int. J. Hydrogen Energy* **43**, 1702–1708 (2017).
81. Jiang, L., Lu, Y., Wu, W., Cao, Z. & Li, T. Microstructure and Mechanical Properties of a CoFeNi 2 V 0.5 Nb 0.75 Eutectic High Entropy Alloy in As-cast and Heat-treated Conditions. *J. Mater. Sci. Technol.* **32**, 245–250 (2016).
82. Gao, M. C., Zhang, B., Guo, S. M., Qiao, J. W. & Hawk, J. A. High-Entropy Alloys in Hexagonal Close-Packed Structure. *Metall. Mater. Trans. A Phys. Metall. Mater. Sci.* **47**, 3322–3332 (2016).
83. Singh, A. K. & Subramaniam, A. Thermodynamic Rationalization of the Microstructures of CrFeNi & CuCrFeNi Alloys. *Adv. Mater. Res.* **585**, 3–7 (2012).
84. Jo, Y. H. *et al.* Utilization of brittle σ phase for strengthening and strain hardening in ductile VCrFeNi high-entropy alloy. *Mater. Sci. Eng. A* **743**, 665–674 (2019).
85. Jiang, H. *et al.* Effects of Tungsten on Microstructure and Mechanical Properties of CrFeNiV0.5Wx and CrFeNi2V0.5Wx High-Entropy Alloys. *J. Mater. Eng. Perform.* **24**, 4594–4600 (2015).
86. Fazakas, É. *et al.* Int . Journal of Refractory Metals and Hard Materials Experimental and theoretical study of Ti 20 Zr 20 Hf 20 Nb 20 X 20 (X = V or Cr) refractory high-entropy alloys. *RMHM* **47**, 131–138 (2014).
87. Mu, Y. *et al.* An ab initio and experimental studies of the structure, mechanical parameters and state density on the refractory high-entropy alloy systems. *J. Alloys Compd.* **714**, 668–680 (2017).
88. Zhang, B., Gao, M. C., Zhang, Y. & Guo, S. M. Senary refractory high-entropy alloy CrxMoNbTaVW. *Calphad Comput. Coupling Phase Diagrams Thermochem.* **51**, 193–201 (2015).
89. Ahmed, O., Lee, J., Mo, H. & Jin, H. The effect of Ti on the sintering and mechanical properties of refractory high-entropy alloy Ti x WTaVCr fabricated via spark plasma sintering for fusion plasma-facing materials. *Mater. Chem. Phys.* **210**, 87–94 (2018).
90. Feuerbacher, M., Heidelmann, M. & Thomas, C. Hexagonal High-entropy Alloys. *Mater. Res. Lett.* **3**, 1–6 (2014).
91. Vrtnik, S. *et al.* Magnetic phase diagram and magnetoresistance of Gd–Tb–

Dy–Ho–Lu hexagonal high-entropy alloy. *Intermetallics* **105**, 163–172 (2019).
92. Liu, Y. *et al.* Microstructure and mechanical properties of refractory HfMo0.5NbTiV0.5Sixhigh-entropy composites. *J. Alloys Compd.* **694**, 869–876 (2017).
93. Tseng, K. K. *et al.* Effects of Mo, Nb, Ta, Ti, and Zr on mechanical properties of equiatomic Hf-Mo-Nb-Ta-Ti-Zr alloys. *Entropy* **21**, 1–14 (2019).
94. Gao, M. C. *et al.* Design of Refractory High-Entropy Alloys. *Jom* **67**, 2653–2669 (2015).
95. Juan, C. *et al.* Solution strengthening of ductile refractory HfMo x NbTaTiZr high-entropy alloys. *Mater. Lett.* **175**, 284–287 (2016).
96. Juan, C. C. *et al.* Enhanced mechanical properties of HfMoTaTiZr and HfMoNbTaTiZr refractory high-entropy alloys. *Intermetallics* **62**, 76–83 (2015).
97. Guo, N. N. *et al.* Intermetallics Microstructure and mechanical properties of in-situ MC-carbide matrix alloy composite. **69**, 74–77 (2016).
98. Chen, Y. *et al.* The microstructure and mechanical properties of refractory high-entropy alloys with high plasticity. *Materials (Basel).* **11**, (2018).
99. Guo, N. N. *et al.* Microstructure and mechanical properties of refractory MoNbHfZrTi high-entropy alloy. *Mater. Des.* **81**, 87–94 (2015).
100. Lu, Y. *et al.* A promising new class of irradiation tolerant materials: Ti 2 ZrHfV 0.5 Mo 0.2 high-entropy alloy. *J. Mater. Sci. Technol.* **35**, 369–373 (2019).
101. Gao, M. C., Zhang, B., Yang, S. & Guo, S. M. Senary Refractory High-Entropy Alloy HfNbTaTiVZr. *Metall. Mater. Trans. A Phys. Metall. Mater. Sci.* **47**, 3333–3345 (2016).
102. Veselý, J. *et al.* Alloy design for intrinsically ductile refractory high-entropy alloys. *J. Appl. Phys.* **120**, 164902 (2016).
103. Podolskiy, A. V *et al.* Materials Science & Engineering A Mechanical properties and thermally activated plasticity of the Ti 30 Zr 25 Hf 15 Nb 20 Ta 10 high entropy alloy at temperatures 4 . 2 – 350 K. *Mater. Sci. Eng. A* **710**, 136–141 (2018).
104. Feuerbacher, M., Heidelmann, M. & Thomas, C. Plastic deformation properties of Zr – Nb – Ti – Ta – Hf high-entropy alloys. 37–41 (2015). doi:10.1080/14786435.2015.1028506
105. Lilensten, L. *et al.* Design and tensile properties of a bcc Ti-rich high-entropy alloy with transformation-induced plasticity. **3831**, (2017).
106. Stepanov, N. D., Yurchenko, N. Y., Zherebtsov, S. V., Tikhonovsky, M. A. & Salishchev, G. A. Aging behavior of the HfNbTaTiZr high entropy alloy. *Mater. Lett.* **211**, 87–90 (2018).
107. Chen, S. Y. *et al.* Phase transformations of HfNbTaTiZr high-entropy alloy at intermediate temperatures. *Scr. Mater.* **158**, 50–56 (2019).
108. Guo, J. *et al.* Robust zero resistance in a superconducting high-entropy alloy at pressures up to 190 GPa. *Proc. Natl. Acad. Sci.* **114**, 13144–13147 (2017).
109. Maiti, S. & Steurer, W. Acta Materialia Structural-disorder and its effect on

mechanical properties in single- phase TaNbHfZr high-entropy alloy. *Acta Mater.* **106**, 87–97 (2016).

110. Karlsson, D. *et al.* Structure and Hydrogenation Properties of a HfNbTiVZr High-Entropy Alloy. *Inorg. Chem.* **57**, 2103–2110 (2018).
111. Pacheco, V. *et al.* Thermal Stability of the HfNbTiVZr High-Entropy Alloy. *Inorg. Chem.* **58**, 811–820 (2019).
112. Guo, W. *et al.* Local atomic structure of a high-entropy alloy: An X-Ray and neutron scattering study. *Metall. Mater. Trans. A Phys. Metall. Mater. Sci.* **44**, 1994–1997 (2013).
113. Huang, H. *et al.* Phase-Transformation Ductilization of Brittle High-Entropy Alloys via Metastability Engineering. **1701678**, 1–7 (2017).
114. Fan, Z., Wang, H., Wu, Y., Liu, X. & Lu, Z. Thermoelectric performance of PbSnTeSe high-entropy alloys. *Mater. Res. Lett.* **5**, 187–194 (2017).
115. Yao, H. W. *et al.* Mechanical properties of refractory high-entropy alloys: Experiments and modeling. *J. Alloys Compd.* **696**, 1139–1150 (2017).
116. Zhang, B., Gao, M. C., Zhang, Y., Yang, S. & Guo, S. M. Senary refractory high entropy alloy MoNbTaTiVW. *Mater. Sci. Technol.* **31**, 1207–1213 (2015).
117. Han, Z. D. *et al.* Intermetallics Effect of Ti additions on mechanical properties of NbMoTaW and VNbMoTaW refractory high entropy alloys. *Intermetallics* **84**, 153–157 (2017).
118. Han, Z. D. *et al.* Microstructures and mechanical properties of TixNbMoTaW refractory high-entropy alloys. *Mater. Sci. Eng. A* **712**, 380–385 (2018).
119. Wang, S. & Xu, J. TiZrNbTaMo high-entropy alloy designed for orthopedic implants : As-cast microstructure and mechanical properties. *Mater. Sci. Eng. C* **73**, 80–89 (2017).
120. Todai, M. *et al.* Scripta Materialia Novel TiNbTaZrMo high-entropy alloys for metallic biomaterials. *SMM* **129**, 65–68 (2017).
121. Yao, H. *et al.* MoNbTaV medium-entropy alloy. *Entropy* **18**, 1–15 (2016).
122. Senkov, O. N., Wilks, G. B., Miracle, D. B., Chuang, C. P. & Liaw, P. K. Refractory high-entropy alloys. *Intermetallics* **18**, 1758–1765 (2010).
123. Wu, Y. D. *et al.* Materials & Design Phase composition and solid solution strengthening effect in TiZrNbMoV high-entropy alloys. **83**, 651–660 (2015).
124. Yao, H. W. *et al.* NbTaV-(Ti,W) refractory high-entropy alloys: Experiments and modeling. *Mater. Sci. Eng. A* **674**, 203–211 (2016).
125. Wang, R. *et al.* Novel metastable engineering in single-phase high-entropy alloy. *Mater. Des.* **162**, 256–262 (2019).
